# Effect of multidisciplinary care on diabetic kidney disease: a retrospective cohort study

**DOI:** 10.1186/s12882-024-03550-w

**Published:** 2024-03-25

**Authors:** Ayano Hayashi, Kayoko Mizuno, Kanna Shinkawa, Kazunori Sakoda, Satomi Yoshida, Masato Takeuchi, Motoko Yanagita, Koji Kawakami

**Affiliations:** 1 Department of Pharmacoepidemiology, Graduate School of Medicine and Public Health, Kyoto University, Yoshida-Konoe-Cho, Sakyo-Ku, Kyoto, 606-8501 Japan; 2 Department of Nephrology, Graduate School of Medicine, Kyoto University, Kyoto, Japan; 3 Graduate School of Public Health, Shizuoka Graduate University of Public Health, Shizuoka, Japan; 4 Institute for the Advanced Study of Human Biology (WPI-ASHBi), Kyoto University, Kyoto, Japan

**Keywords:** Diabetic kidney disease, Multidisciplinary care, Retrospective cohort study

## Abstract

**Background:**

Diabetic kidney disease (DKD) is the most common disease among patients requiring dialysis for the first time in Japan. Multidisciplinary care (MDC) may prevent the progression of kidney failure. However, the effectiveness and timing of MDC to preserve kidney function in patients with DKD is unclear. Therefore, the aim of this study was to investigate whether MDC for patients with DKD affects the preservation of kidney function as well as the timing of MDC in clinical practice.

**Methods:**

In this retrospective cohort study, we identified patients with type 2 diabetes mellitus and DKD from April 2012 to January 2020 using a nationwide Japanese healthcare record database. The fee code for medical guidance to prevent dialysis in patients with diabetes was used to distinguish between the MDC and non-MDC groups. The primary outcome was a 40% decline in the estimated glomerular filtration rate, and secondary outcomes were death, hospitalization, permanent dialysis, kidney failure with replacement therapy, and emergency temporary catheterization. Propensity score matching was performed, and Kaplan–Meier and multivariable Cox regression analyses were performed.

**Results:**

Overall, 9,804 eligible patients met the inclusion criteria, of whom 5,614 were matched for the main analysis: 1,039 in the MDC group, and 4,575 in the non-MDC group. The primary outcome did not differ between the groups (hazard ratio: 1.18, [95% confidence interval: 0.99–1.41], *P* = 0.07). The groups also did not differ in terms of the secondary outcomes. Most patients with DKD received their first MDC guidance within 1 month of diagnosis, but most received guidance only once per year.

**Conclusions:**

Although we could not demonstrate the effectiveness of MDC on kidney function in patients with DKD, we clarified the characteristics of such patients assigned the fee code for medical guidance to prevent dialysis related to diabetes.

**Supplementary Information:**

The online version contains supplementary material available at 10.1186/s12882-024-03550-w.

## Introduction

Among patients with chronic kidney disease (CKD), the number of those requiring dialysis is increasing. Currently, the number of patients receiving kidney replacement therapy (KRT) globally exceeds 2.5 million [1]. In Japan, the number of patients who require dialysis has been steadily increasing since the 1980s, to a total of approximately 340,000 by 2020 [2], amounting to an estimated medical cost of approximately 14 billion US dollars [3]. Patients with diabetic kidney disease (DKD) account for the largest proportion (40%) of those requiring dialysis [2]. Several lifestyle habits contribute to the progression of CKD [4–6]. However, as patients with CKD tend to experience few symptoms until nephropathy progresses, they may not be aware of the need to improve their lifestyle. Multidisciplinary care (MDC) has attracted considerable attention in recent years and has been mentioned in the Kidney Disease: Improving Global Outcomes guidelines [7]. An MDC team is composed of doctors and other healthcare providers, such as nurses, dietitians, and medical social workers, all of whom raise patients’ awareness of CKD risk factors [8].

Several observational studies have demonstrated the effectiveness of MDC in preserving kidney function in patients with CKD [9–12]. However, few studies have demonstrated the effectiveness of MDC for patients with DKD, [13, 14] while another study revealed no significant difference between MDC and normal intervention [15]. Several single-center [14, 16–18] and multicenter studies [19, 20] on MDC have been reported in Japan. In those studies, MDC was provided at a different time for each patient. In the only multicenter randomized clinical trial conducted in Japan on patients with type 2 diabetes mellitus (T2DM) with overt nephropathy, the difference between MDC and conventional treatment was not statistically significant [21]. All abovementioned studies had certain limitations, such as low power due to the small number of study patients or an insufficient observation period to capture changes in kidney prognosis. Therefore, epidemiological studies on kidney prognosis among patients with DKD receiving MDC are warranted in Japan.

In this study, we aimed to investigate whether MDC affects the preservation of kidney function in patients with DKD in Japan as well as the timing of MDC for such patients in clinical practice.

## Materials and methods

### Study design and setting

For this retrospective cohort study, we used data from the RWD database, which is maintained by the Health, Clinic, and Education Information Evaluation Institute, with support from Real World Data Co., Ltd [22, 23]. It contained records of 24 million patients from 225 medical institutions across Japan as of 2022. Those hospitals included various types of hospitals, private and public, from large medical centers to clinics [24]. The database includes the following patient information: demographic data, diagnoses according to the International Classification of Diseases, 10th Revision (ICD-10) codes, procedures, medications, and laboratory test results. This study was approved by the Ethics Committee of Kyoto University (R3141) and did not require individual consent because data were anonymized. During the study, we adhered to the tenets of the Declaration of Helsinki.

### Patients’ criteria

We extracted data of patients with diagnostic codes for DKD (E112 and E142) [25] for whom diagnostic codes for T2DM (E11–E14) had been assigned before the index date (date when the diagnostic codes for DKD were assigned). Other inclusion criteria were age ≥ 20 years at index date and an index estimated glomerular filtration rate (eGFR; defined as the eGFR measured close to the index date [within 90 days]) of 15–90 mL/min/1.73 m^2^. We excluded patients without index eGFR data, without available follow-up eGFR data more than 1 year after the index date, who had a diagnostic code for type 1 diabetes (E10) before the index date, who had undergone KRT before or within 1 year of the index date, and in whom the fee code for medical guidance for the prevention of dialysis related to diabetes was assigned before the index date. We used landmark analysis to reduce immortal time bias [26] because the time between DKD diagnosis and guidance to prevent dialysis differed among patients, and no outcome occurred between diagnosis and treatment in certain patients. We defined the landmark time as 1 year after DKD diagnosis, and patients in whom one of the outcomes occurred within 1 year from the index date were excluded from the analysis. The study period for the index date was April 2012 to January 2020. The time window for this study is summarized in Supplemental Fig 1.

### Medical guidance to prevent dialysis in patients with diabetes

We used the fee codes for medical guidance to prevent dialysis in patients with diabetes to distinguish between the MDC and non-MDC groups. The fee code was identified using the procedure code B001-27. First, staff required for assignment of the fee code comprised a team of physicians, nurses, and dietitians with at least 5 years of experience in diabetes or DKD guidance. Nurses with 2 years of experience and at least 1,000 h of guidance were also acceptable. The requirements for the assignment of the fee code are as follows [27]: 1) glycated hemoglobin (HbA1c) level > 6.5% (National Glycohemoglobin Standardization Program value) or use of oral hypoglycemic agents or insulin; 2) DKD stage 2 or higher (microalbuminuria and eGFR ≥ 30 mL/min/1.73 m^2^ before the dialysis initiation); and 3) fee code assigned once a month and not in the same month as that for outpatient nutritional guidance. The need for and specifics of the guidance was left to the clinician’s discretion. Examples of guidance included salt reduction, weight loss, smoking cessation, cessation of excessive alcohol consumption, exercise therapy, KRT, and management of comorbidities. The method by which guidance was provided was as follows. Physicians provide guidance to nurses and dietitians in advance, with each professional creating individualized instructional plans. On the designated guidance day, the physicians, nurses, and dietitians conduct separate guidance sessions and subsequently document each session for later review. Thereafter, team conferences and assessments of instructional effectiveness may be conducted as needed, although the frequency is not explicitly defined and is left to the discretion of each facility**.** Additionally, as a fee code need not be assigned for team conferences, the actual occurrence of team conferences could not be extracted from the database.

The MDC and non-MDC groups included patients who were and were not, respectively, assigned the guidance fee code at least once within 1 year of the index date. This allocation was performed according to an intention-to-treat analysis regardless of fee codes assigned after the landmark time.

### Variables

The primary outcome was a 40% decline in eGFR from the index eGFR (confirmed twice at least 30 days apart). This outcome is in line with the 30%–40% reduction in eGFR over 2–3 years in patients with CKD with rapid progression, serving as a surrogate endpoint for kidney failure with replacement therapy (KFRT) [28, 29]. We calculated the eGFR by using the following well-validated formula proposed by the Japanese Society of Nephrology [30]:

$$\mathrm{eGFR}\;\mathrm{mL}/\min/1.73\;\mathrm m^2\:=\:194\,\:\times\:\,\mathrm{serum}\;\mathrm{creatinine}^{-1.094}\;\mathrm{mg}/\mathrm{dL}\,\:\times\:\,\mathrm{age}^{-0.287}\;\mathrm{years}\,\:\times\:\,0.739\;\mathrm{if}\;\mathrm{female}$$

Secondary outcomes were death from any cause, hospitalization, permanent dialysis, KFRT (eGFR ≤ 15 mL/min/1.73 m^2^, confirmed twice within 30 days), and emergency temporary catheterization for blood access.

Covariates used included age, sex, laboratory data (eGFR, proteinuria, and low-density lipoprotein cholesterol, uric acid, and HbA1c levels), body mass index, smoking status, duration of diabetes (time from date of diagnosis of T2DM to that of DKD), comorbidities (hypertension, hyperlipidemia, ischemic heart disease, and hyperuricemia), medications (oral hypoglycemic agents, insulin, calcium channel blockers, renin–angiotensin system antagonists, β-blockers, lipid-lowering agents, and uric acid-lowering agents), procedures (percutaneous coronary angioplasty, coronary artery bypass grafting, and cerebrovascular surgery), and the number of hospital beds. Covariates were identified by their diagnostic, procedural, and Anatomical Therapeutic Chemical (for medications) codes (Supplemental Table 1). Proteinuria was assessed using a urine dipstick test that is used for universal screening in Japan [31].

### Statistical analysis

Continuous variables are presented as means and standard deviations (SDs) or medians and interquartile ranges (IQR). Categorical variables are presented as frequencies and percentages. Propensity score (PS) matching was used to match patients’ backgrounds between the two groups. Covariates used for PS calculation were sex, age, number of hospital beds, index eGFR, duration of diabetes, medications, and procedures. Other covariates were not used because of missing values, and we believe that the covariates used were sufficient to adjust for confounding factors based on previous studies [18, 32–34]. We used 1:5 matching, a logistic regression model, and the non-replacement nearest-neighbor method with a caliper width of 0.2 of the SD. After PS matching, the absolute standardized mean difference (ASMD) was used to compare the two groups, and Kaplan–Meier survival curves were constructed. Data were censored at death and at the date of the last observation in the database. Time zero was defined as the landmark time, and log-rank tests were used for comparison. Multivariable Cox regression was used to estimate the hazard ratios (HRs) and 95% confidence intervals (CIs). Covariates used for adjustment in the multivariable analysis were the same as those used for PS calculation. All P-values were two-sided, and P < 0.05 was considered statistically significant. All analyses were performed using R version 4.2.3 (R Foundation for Statistical Computing, Vienna, Austria).

### Sensitivity analysis

We performed several sensitivity analyses to confirm the robustness of the results. First, we added an extra criterion for censoring. If patients received their first guidance after the landmark time, they were censored at that time and considered a part of the non-MDC group. Second, we revised the definitions of the MDC and non-MDC groups. The former included patients who had received guidance more than twice within 1 year of the index date, while the latter included patients who had not received or only one guidance during that period because only one guidance might not have been enough. Third, we set the upper limit for the observation period at 3 years after the index date; all patients were censored thereafter. Fourth, we changed the duration of the landmark time from 1 to 2 years. In this analysis, patients without available follow up eGFR data for more than 2 years after the index date or who had received KRT within 2 years of the index date were also excluded, and both groups were reclassified accordingly. Fifth, we conducted inverse probability of treatment weighting (IPTW) and overlap weighting [35] instead of PS matching. The first three sensitivity analyses were performed for the primary and secondary outcomes, whereas the remaining sensitivity analyses were performed only for the primary outcome.

## Results

### Baseline characteristics of the study population

**Fig. 1:**
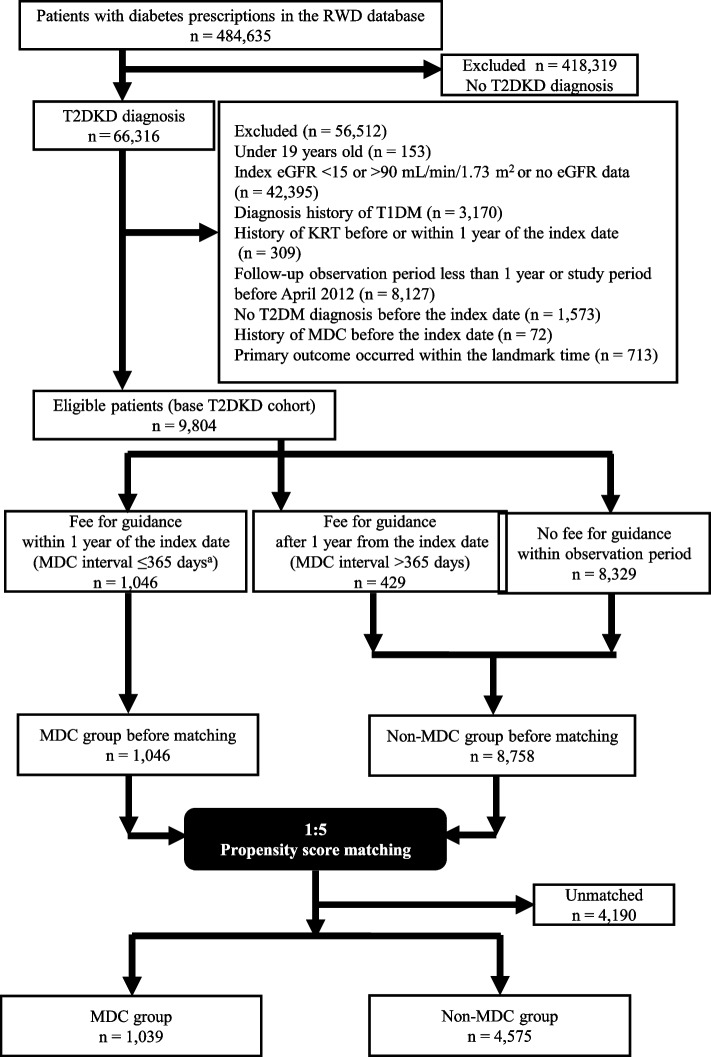
Flow diagram of patients analyzed for the primary outcome. ^a^Interval between DKD diagnosis and first guidance to prevent dialysis for patients with diabetes. RWD: Real World Data, T2DKD: type 2 diabetic kidney disease, eGFR: estimated glomerular filtration rate, T1DM: type 1 diabetes mellitus, KRT: kidney replacement therapy, T2DM: type 2 diabetes mellitus, MDC: multidisciplinary care

We screened 484,635 patients with prescriptions for diabetes (Anatomical Therapeutical Chemical code A10). The number of eligible patients was 9,804, of whom 5,614 were matched for the main analysis: 1,039 in the MDC group, and 4,575 in the non-MDC group (Fig. 1).

A comparison of patients’ backgrounds before matching revealed that approximately 70% of patients in both groups were aged 60–70 years. The duration of diabetes was longer (median: 7.2 vs. 5.1 years), and the index eGFR was slightly lower (median: 56 vs. 61 mL/min/1.73 m^2^), in the MDC group than in the non-MDC group. Patients in the MDC group tended to use more medications and have more comorbidities than those in the non-MDC group. However, in terms of laboratory data and excluding proteinuria, the groups did not significantly differ (Table 1). Almost all patients in the MDC group were PS matched to patients in the non-MDC group. An ASMD of < 0.1 for all variables used for PS calculation indicated that the groups were well balanced (Supplemental figs 2 and 3).

**Table 1:** Baseline characteristics of the study population before and after PS matching for the primary outcome

Variables	Before matching			After matching		
	MDC group	Non-MDC group	SMD	MDC group	Non-MDC group	SMD
	*n* = 1,046	*n* = 8,758		*n* = 1,039	*n* = 4,575	
Variables used for PS calculation						
Age (years), median (IQR)	70 (63–76)	70 (63–77)	0.01	70 (63–76)	70 (63–76)	0.02
Categorized, n (%)			0.12			0.03
20–49	55 (5.3)	511 (5.8)		55 (5.3)	255 (5.6)	
50–59	122 (11.7)	1,076 (12.3)		122 (11.7)	494 (10.8)	
60–69	327 (31.3)	2,700 (30.8)		327 (31.5)	1,460 (31.9)	
70–79	406 (38.8)	3,030 (34.6)		399 (38.4)	1,729 (37.8)	
≥ 80	136 (13.0)	1,441 (16.5)		136 (13.1)	637 (13.9)	
Male sex, n (%)	689 (65.9)	5,654 (64.6)	0.03	683 (65.7)	2,963 (64.8)	0.02
Hospital size by number of beds, n (%)			0.35			0.09
> 500	112 (10.7)	1,836 (21.0)		112 (10.8)	569 (12.4)	
300–499	634 (60.6)	4,051 (46.3)		627 (60.3)	2,555 (55.8)	
100–299	300 (28.7)	2,844 (32.5)		300 (28.9)	1451 (31.7)	
20–99	0 (0.0)	10 (0.1)		0 (0.0)	0 (0.0)	
< 20	0 (0.0)	17 (0.2)		0 (0.0)	0 (0.0)	
Duration of diabetes (years), median (IQR)	7.2 (2.0–13.1)	5.1 (0.7–11.0)	0.22	7.1 (2.0–13.1)	6.4 (1.2–12.0)	0.09
Categorized, n (%)			0.20			0.08
0–5	420 (40.2)	4,332 (49.5)		420 (40.4)	1,991 (43.5)	
5–15	435 (41.6)	3,274 (37.4)		433 (41.7)	1,872 (40.9)	
15–25	170 (16.3)	1,032 (11.8)		166 (16.0)	634 (13.9)	
≥ 25	21 (2.0)	120 (1.4)		20 (1.9)	78 (1.7)	
Index eGFR (ml/min/1.73 m^2^), median (IQR)	56 (43–70)	61 (45–74)	0.16	57(43–70)	57(43–71)	0.03
Categorized, n (%)			0.22			0.02
G2: 60–90	443 (42.4)	4,604 (52.6)		443 (42.6)	1,987 (43.4)	
G3a: 45–60	311 (29.7)	2,014 (23.0)		308 (29.6)	1,341 (29.3)	
G3b: 30–45	190 (18.2)	1,299 (14.8)		186 (17.9)	811 (17.7)	
G4: 15–30	102 (9.8)	841 (9.6)		102 (9.8)	436 (9.5)	
Medication use, n (%)						
Oral hypoglycemic agents	847 (81.0)	5,726 (65.4)	0.36	840 (80.8)	3,593 (78.5)	0.06
Insulin	306 (29.3)	2,019 (23.1)	0.14	302 (29.1)	1,209 (26.4)	0.06
Ca blockers	454 (43.4)	2,762 (31.5)	0.25	449 (43.2)	1,879 (41.1)	0.04
RAS-antagonists	588 (56.2)	3,523 (40.2)	0.32	581 (55.9)	2,426 (53.0)	0.06
β-blockers	174 (16.6)	1,026 (11.7)	0.14	170 (16.4)	690 (15.1)	0.04
Lipid-lowering agents	512 (48.9)	3,375 (38.5)	0.21	506 (48.7)	2,140 (46.8)	0.04
Uric acid-lowering agents	172 (16.4)	920 (10.5)	0.18	167 (16.1)	673 (14.7)	0.04
Procedure, n (%)						
Percutaneous coronary intervention	61 (5.8)	202 (2.3)	0.18	54 (5.2)	164 (3.6)	0.08
Coronary artery bypass grafting	3 (0.3)	11 (0.1)	0.04	3 (0.3)	9 (0.2)	0.02
Cerebral vascular surgery	1 (0.1)	3 (0.0)	0.02	1 (0.1)	3 (0.1)	0.01
Variables not used for PS calculation						
BMI (kg/m^2^), mean (SD)	25.5 (4.4)	24.9 (4.6)	0.14	25.6 (4.4)	25.1 (4.5)	0.11
Categorized, n (%)			0.35			0.30
< 18.5	14 (1.3)	107 (1.2)		14 (1.3)	48 (1.0)	
18.5–25.0	173 (16.5)	937 (10.7)		169 (16.3)	508 (11.1)	
≥ 25.0	196 (18.7)	828 (9.5)		194 (18.7)	490 (10.7)	
Missing data	663 (63.4)	6,886 (78.6)		662 (63.7)	3,529 (77.1)	
Smoking, n (%)			0.31			0.27
Non-smoker	214 (20.5)	1,012 (11.6)		210 (20.2)	556 (12.2)	
Past or current smoker	146 (14.0)	811 (9.3)		144 (13.9)	467 (10.2)	
Missing data	686 (65.6)	6,935 (79.2)		685 (65.9)	3,552 (77.6)	
HbA1c (%), median (IQR)	7.2 (6.6–7.9)	7.1 (6.5– 8.0)	0.02	7.2 (6.6–7.9)	7.1 (6.5–8.1)	0.02
Categorized, n (%)			0.26			0.25
< 6.0	37 (3.5)	772 (8.8)		37 (3.6)	389 (8.5)	
6.0–7.0	369 (35.3)	3,136 (35.8)		366 (35.2)	1,622 (35.5)	
7.0–8.0	375 (35.9)	2,470 (28.2)		373 (35.9)	1,305 (28.5)	
≥ 8.0	252 (24.1)	2,249 (25.7)		251 (24.2)	1,199 (26.2)	
Missing data	13 (1.2)	131 (1.5)		12 (1.2)	60 (1.3)	
Proteinuria^a^, n (%)			0.39			0.31
-	285 (27.2)	3,903 (44.6)		285 (27.4)	1,855 (40.5)	
±	212 (20.3)	1,352 (15.4)		212 (20.4)	711 (15.5)	
1 +	196 (18.7)	1,094 (12.5)		195 (18.8)	640 (14.0)	
2 +	128 (12.2)	802 (9.2)		128 (12.3)	436 (9.5)	
3 +	66 (6.3)	563 (6.4)		66 (6.4)	329 (7.2)	
4 +	6 (0.6)	83 (0.9)		6 (0.6)	46 (1.0)	
Missing data	153 (14.6)	961 (11.0)		147 (14.1)	558 (12.2)	
LDL-C (mg/dl), mean (SD)	103 (32)	108 (33)	0.15	103 (32)	106 (33)	0.09
Categorized, n (%)			0.14			0.09
< 100	398 (38.0)	3,023 (34.5)		398 (38.3)	1,679 (36.7)	
100–120	180 (17.2)	1,657 (18.9)		180 (17.3)	854 (18.7)	
120–140	124 (11.9)	1,197 (13.7)		123 (11.8)	585 (12.8)	
140–160	51 (4.9)	613 (7.0)		51 (4.9)	289 (6.3)	
≥ 160	43 (4.1)	431 (4.9)		43 (4.1)	202 (4.4)	
Missing data	250 (23.9)	1,837 (21.0)		244 (23.5)	966 (21.1)	
Uric acid (mg/dl), mean (SD)	5.9 (1.6)	6.1 (4.2)	0.06	5.9 (1.7)	6.2 (4.1)	0.09
Categorized, n (%)			0.12			0.12
< 7.0	776 (74.2)	6,368 (72.7)		771 (74.2)	3,275 (71.6)	
7.0–8.0	123 (11.8)	855 (9.8)		121 (11.6)	464 (10.1)	
8.0–9.0	38 (3.6)	318 (3.6)		38 (3.7)	179 (3.9)	
≥ 9.0	32 (3.1)	311 (3.6)		32 (3.1)	182 (4.0)	
Missing data	77 (7.4)	906 (10.3)		77 (7.4)	475 (10.4)	
Comorbidities, n (%)						
Hypertension	860 (82.2)	6,308 (72.0)	0.24	853 (82.1)	3,616 (79.0)	0.08
Hyperlipidemia	777 (74.3)	5,654 (64.6)	0.21	771 (74.2)	3,144 (68.7)	0.12
Ischemic heart disease	433 (41.4)	3,047 (34.8)	0.14	426 (41.0)	1,724 (37.7)	0.07
Hyperuricemia	220 (21.0)	1,317 (15.0)	0.16	215 (20.7)	834 (18.2)	0.06
Observation period^b^ (years), median (IQR)	3.5 (2.1–4.9)	4.0 (2.6–5.9)	0.30	3.5 (2.2–4.9)	3.9 (2.6–5.9)	0.30

*PS* Propensity score, *MDC* Multidisciplinary care, *IQR* Interquartile range, *eGFR* Estimated glomerular filtration rate, *Ca* Calcium channel, *RAS* Renin–angiotensin system, *BMI* Body mass index, *SD* Standard deviation, *HbA1c* Glycated hemoglobin, *LDL-C* Low-density lipoprotein cholesterol, *SMD* Standardized mean difference

^a^Urine dipstick evaluation

^b^From the index date to the date of the last observation in the database

We excluded patients in whom each outcome occurred within 1 year of the index date; therefore, the number of eligible patients differed for each outcome (Supplemental Table 2 and Supplemental Fig 4).

**Table 2:** Frequencies of events and hazard ratios for primary and secondary outcomes in the main analysis

Outcome	Events	Patients	Person-days	Incidence rate^a^ (95% CI)	Crude HR (95% CI)	Adjusted HR (95% CI)
**40% eGFR decline**						
Non-MDC group	679	4,575	5,005,934	1.36 (1.26–1.46)	Ref	Ref
MDC group	150	1,039	921,928	1.63 (1.38–1.91)	1.16 (0.97–1.39)	1.18 (0.99–1.41)
**Death**						
Non-MDC group	354	4,904	5,940,089	0.60 (0.54–0.66)	Ref	Ref
MDC group	55	1,090	1,084,772	0.51 (0.38–0.66)	0.86 (0.65–1.15)	0.89 (0.66–1.18)
**Permanent dialysis**						
Non-MDC group	228	4,904	5,785,900	0.39 (0.34–0.45)	Ref	Ref
MDC group	34	1,090	1,063,035	0.32 (0.22–0.45)	0.80 (0.56–1.15)	0.85 (0.59–1.22)
**Hospitalization**						
Non-MDC group	1,417	3,541	3,560,577	3.98 (3.78–4.19)	Ref	Ref
MDC group	302	840	667,727	4.52 (4.03–5.06)	1.13 (0.99–1.28)	1.10 (0.97–1.24)
**Temporary catheterization**						
Non-MDC group	10	4,904	5,932,936	0.02 (0.01–0.03)	Ref	Ref
MDC group	2	1,090	1,084,558	0.02 (0.002–0.07)	1.13 (0.25–5.19)	1.12 (0.24–5.22)
**KFRT**						
Non-MDC group	308	4,695	5,490,417	0.56 (0.50–0.63)	Ref	Ref
MDC group	65	1,062	1,023,052	0.64 (0.49–0.81)	1.08 (0.83–1.42)	1.10 (0.84–1.44)

The adjusted models included adjustments for sex, age (categorized), number of hospital beds, eGFR (categorized), duration of diabetes (categorized), medication use, and procedures

*MDC* Multidisciplinary care, *HR* Hazard ratio, *CI* Confidence interval, *eGFR* Estimated glomerular filtration rate, *KFRT* Kidney failure with replacement therapy

^a^Incidence rate per 10,000 person-days

### Main analysis

**Fig. 2:**
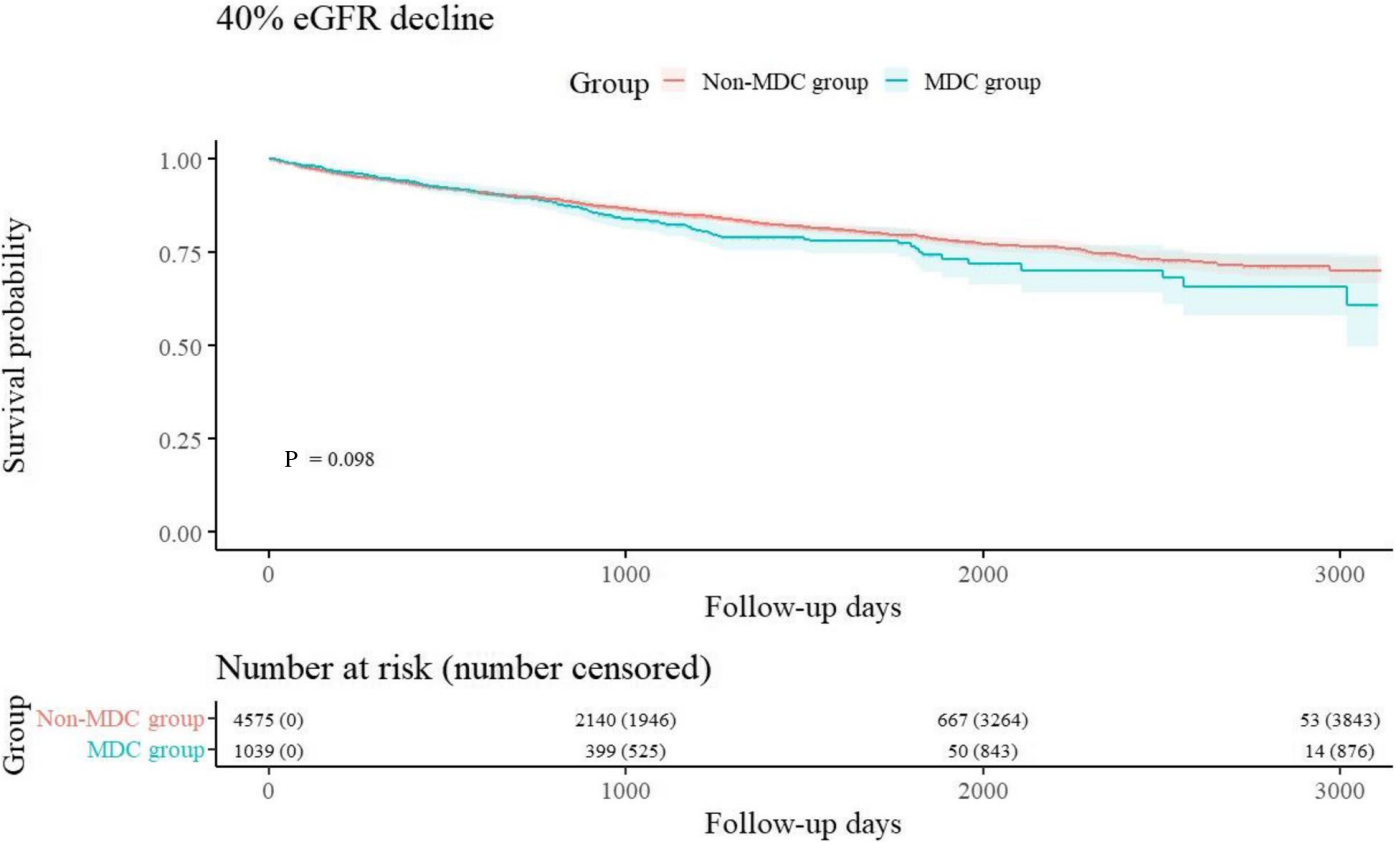
Kaplan–Meier curves for the main analysis of the primary outcome (40% decline in eGFR)

**Fig. 3:**
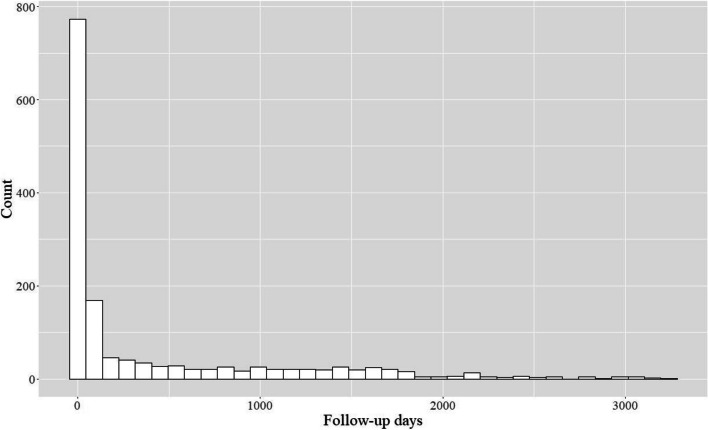
Interval between the diagnosis of diabetic kidney disease and the first guidance

**Fig. 4:**
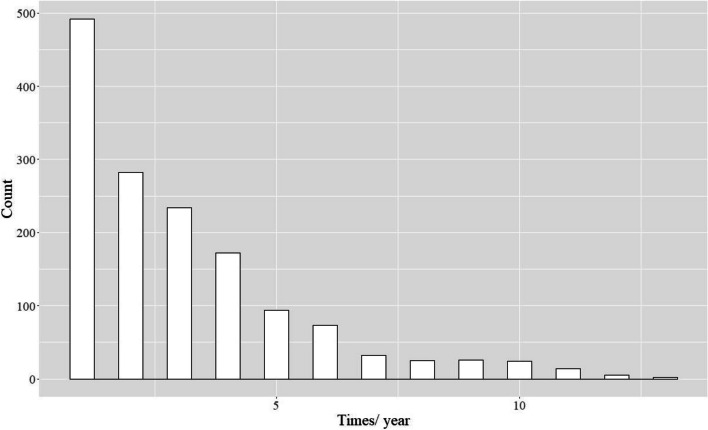
Maximum instances of guidance to prevent dialysis in patients with diabetes per year

We plotted the Kaplan–Meier curves of the primary (Fig. 2) and secondary (Supplemental Fig. 5) outcomes. During the observation period, the primary outcome occurred in 150/1,039 patients in the MDC group (14%) and 679/4,575 patients in the non-MDC group (15%). The log-rank test for the primary outcome revealed no difference between the groups (*P* = 0.098), nor did those for the secondary outcomes. The groups also did not significantly differ both in the primary outcome according to multivariable Cox regression analysis (adjusted HR: 1.18 [95% CI: 0.99–1.41], *P* = 0.07) and in the secondary outcomes (Table 2). As for the calculation of the fee for medical guidance to prevent dialysis related to diabetes, most of the patients were charged within 1 month of the index date (median: 29.0 days, IQR: 0–560.5 days) (Fig. 3), and most received guidance only once (median: 2.0 times, IQR: 1.0–4.0 times) (Fig. 4).

### Sensitivity analysis

Although the results of sensitivity analyses were mostly consistent with those of our main analysis, the HR for the primary outcome was higher in the MDC group than in the non-MDC group when the definition of censoring was changed (adjusted HR: 1.21 [95% CI: 1.01–1.44], *P* = 0.04), the definition of landmark time was changed (adjusted HR: 1.41 [95% CI: 1.14–1.73], *P* < 0.001), and when IPTW was used instead of PS matching (adjusted HR: 1.24 [95% CI: 1.00–1.53], *P* = 0.05) (Table 3). Moreover, the HR for death was lower in the MDC group than in the non-MDC group when the upper limit of the observation period was set at 3 years (adjusted HR: 0.68 [95% CI: 0.47–0.98], *P* = 0.04) (Supplemental Table 3).

**Table 3:** Frequency of events and hazard ratio for the primary outcome in sensitivity analyses

Outcome: 40% eGFR decline	Events	Patients	Person-days	Incidence rate^a^ (95% CI)	Adjusted HR (95% CI)
**Main analysis**					
Non-MDC group	679	4,575	5,005,934	1.36 (1.26–1.46)	Ref
MDC group	150	1,039	921,928	1.63 (1.38–1.91)	1.18 (0.99–1.41)
**Changing the definition of censoring**					
Non-MDC group	630	4,575	4,778,767	1.32 (1.22–1.43)	Ref
MDC group	150	1,039	921,928	1.63 (1.38–1.91)	1.21 (1.01–1.44)
**Changing the definition of the MDC and non-MDC groups**					
Non-MDC group	511	3,349	3,665,556	1.39 (1.28–1.52)	Ref
MDC group	95	689	590,790	1.61 (1.30–1.97)	1.18 (0.95–1.47)
**Setting an upper limit for the observation period of 3 years**					
Non-MDC group	519	4,575	3,513,034	1.48 (1.35–1.61)	Ref
MDC group	123	1,039	735,007	1.67 (1.39–2.00)	1.14 (0.94–1.39)
**Changing the definition of landmark time to 2 years**					
Non-MDC group	440	3,661	3,700,736	1.19 (1.08–1.31)	Ref
MDC group	114	821	663,891	1.72 (1.42–2.06)	1.41 (1.14–1.73)
**IPTW**					
Non-MDC group	1,258	8,758	9,635,373	1.31 (1.23–1.38)	Ref
MDC group	151	1,046	925,496	1.63 (1.38–1.91)	1.24 (1.00–1.53)
**Overlap weighting**					
Non-MDC group	1,258	8,758	9,635,373	1.31 (1.23–1.38)	Ref
MDC group	151	1,046	925,496	1.63 (1.38–1.91)	1.13 (0.94–1.35)

The adjusted models included adjustments for sex, age (categorized), number of hospital beds, eGFR (categorized), duration of diabetes (categorized), medication use, and procedures

*MDC* Multidisciplinary care, *HR* Hazard ratio, *CI* Confidence interval, *eGFR* Estimated glomerular filtration rate, *IPTW* Inverse probability of treatment weighting

^a^Incidence rate per 10,000 person-days

## Discussion

In this retrospective cohort study of 5,614 patients from a nationwide database, we discovered no significant differences in any outcomes between the MDC and non-MDC groups. We also discovered that most patients who were diagnosed with DKD received their first medical guidance to prevent dialysis within 1 month of diagnosis; however, most received only one guidance per year.

The effectiveness of MDC for DKD in terms of kidney outcomes remains controversial. In one study, MDC in the DKD clinic was associated with a lower risk of progression to KFRT compared to care in a non-DKD clinic (adjusted HR: 0.55 [95% CI: 0.36–0.83], *P* = 0.004) [13]. However, that was a case–control study nested in a population of patients with DKD at a secondary diabetes care center, and the CKD stage of their study sample was only stage G3 or G4 (eGFR: 15–59 mL/min/1.73 m^2^), limiting the generalizability of their results to the broader population of patients with DKD. In a multicenter, randomized, controlled trial with a 5-year follow-up period, intensive team-based treatment for patients with DKD did not significantly reduce the risk of a primary composite outcome (KFRT, doubling of serum creatinine concentration, or death from any cause) compared with conventional treatment (adjusted HR: 0.69 [95% CI: 0.43–1.11], *P* = 0.13) [21]. The study sample had a median diabetes duration of approximately 15 years and a median eGFR of 40 mL/min/1.73 m^2^. In comparison, our study sample had a median diabetes duration of 6.5 years and a median eGFR of 57 mL/min/1.73 m^2^. This means that patients in the previous study had more advanced nephropathy than those in our study.

Several of our results are worth discussing. First, we are not aware of previous multicenter studies in Japan wherein the fee for medical guidance to prevent dialysis in patients with diabetes was calculated. We did investigate this factor for patients in whom the fee was calculated after the diagnosis of DKD. In previous studies, such guidance was provided every 2–3 months, [10, 11, 14, 21, 36] which is more frequent than that in our study (mostly once a year), although guidance in our study was provided shortly after diagnosis. We believe that the frequency of MDC guidance in our study was inadequate, which might have led to the lack of differences in outcomes between the two groups. Second, we clarified the characteristics of patients who received guidance after being diagnosed with DKD. Patients in the MDC group tended to use more medications and have more comorbidities than those in the non-MDC group, similar to those in previous studies [10, 12]. Therefore, medication use and comorbidities of patients with DKD may factor into a clinician’s decision for MDC guidance. Third, the observation that the HR for the primary outcome was higher in the MDC group in some sensitivity analyses may be interpreted in light of the higher frequency of eGFR measurements in the MDC group, which, in turn, can be explained by the higher frequency of hospital visits in the MDC group (Supplemental Table 4). On the other hand, we considered that, in the sensitivity analysis in which we set the upper limit for the observation period to 3 years, the observation that the HR for death was lower in the MDC group suggested the potential effectiveness of MDC in improving short-term survival.

The fee for medical guidance to prevent dialysis in patients with diabetes was originally introduced as part of the medical policy in Japan in 2012 to reduce the annual number of patients requiring dialysis owing to diabetic nephropathy [27, 37]. We believe that the effectiveness of that initiative in delaying the progression of nephropathy should be evaluated. However, this fee has been incorporated into only a few studies [38, 39]. To our knowledge, our study was the first wherein the MDC guidance fee code was evaluated in a real-world database; however, we could not demonstrate its effectiveness on kidney outcomes. As discussed earlier, this might have been due to the low frequency of such guidance provided in this study. In the future, qualitative studies should be conducted to verify whether such guidance is effective in specific contexts.

Our study had several limitations. First, we could not assess the content of the MDC guidance. However, the educational content is standardized according to guidelines of the Japan Diabetes Society and the Japanese Society of Nephrology, and we believe that the medical fee code accurately reflected MDC guidance in this study and ensured the reproducibility of the research. Second, this was a retrospective cohort study; thus, we are aware of several unmeasured confounding factors, such as the relationship between patients and the multidisciplinary team staff and the adherence of patients to therapeutic indications. We might have substantially overestimated the effectiveness of MDC, as patients who tend to strictly adhere to guidance may be more likely to undergo MDC, leading to a potentially lower incidence of kidney function decline. We also did not address information bias due to the number of eGFR measurements, as mentioned above, or the occurrence of comorbidities after the index date, which might have affected each outcome. Further accumulation of evidence is desirable while considering these factors in the study design. Third, we did not consider the impact of nutritional guidance on diabetes before the diagnosis of DKD. However, almost all patients in the RWD database were assigned a fee code for outpatient nutritional guidance regardless of being assigned a fee code for medical guidance to prevent dialysis owing to diabetes (Supplemental Table 5). Thus, most patients in our study likely received nutritional guidance in the past. Fourth, the accuracy of the ICD-10 code for DKD has not been validated in Japan, but the validity was likely high in our study because we selected patients with a past diagnosis of T2DM. Finally, the fee for medical guidance to prevent dialysis in patients with diabetes is Japan-specific; therefore, our results may not be applicable to other countries.

In conclusion, patients receiving MDC did not differ from those receiving conventional care in terms of a 40% decline in eGFR. We clarified the characteristics of patients assigned with the fee code for medical guidance to prevent dialysis related to diabetes, such as when the fee code was assigned relative to the diagnosis of DKD in Japan, by using a nationwide database. Further accumulation of studies is desirable considering the limitations of our study design while considering factors such as the content of the medical guidance provided.

### Supplementary Information

**Supplementary Material 1.**

## Data Availability

The datasets generated and/or analyzed during the current study are not publicly available due to the policy of the institution but are available from the corresponding author on reasonable request with permission of Real World Data Co., Ltd.
