# Supplementary material for: Effect of multidisciplinary care on diabetic kidney disease: a retrospective cohort study
Source: BMC Nephrol. 2024 Mar 25;25:114. doi: 10.1186/s12882-024-03550-w (PMC10962205; doi:10.1186/s12882-024-03550-w)
Supplement: Supplementary file 1 — Supplementary Material 1. [file 12882_2024_3550_MOESM1_ESM.docx]

**Supplemental Material**

Supplemental Table 1: ICD-10, procedural, and ATC for medications used to identify covariates codes

Supplemental Table 2: Baseline characteristics of the study population before and after propensity score matching for secondary outcomes: (A) death, permanent dialysis, temporary catheterization, (B) hospitalization, and (C) KDRT

Supplemental Table 3: Frequency of events and hazard ratios (HRs) for secondary outcomes in the sensitivity analyses compared with the main analysis

Supplemental Table 4: Comparison of the number of eGFR measurements between the MDC and non-MDC groups for the primary outcome

Supplemental Table 5: Evaluation of outpatient nutritional guidance for all patients in the RWD database

Supplemental Figure 1: The time window for this study

Supplemental Figure 2: Distribution of propensity scores before and after matching

Supplemental Figure 3: Covariate balance in propensity score matching

Supplemental Figure 4: Flow diagrams for the secondary outcomes: (A) death, permanent dialysis, temporary catheterization, (B) hospitalization, and (C) KFRT

Supplemental Figure 5: Kaplan–Meier curves for the main analysis of secondary outcomes: (A) death, (B) permanent dialysis, (C) hospitalization, (D) temporary catheterization, and (E) KFRT

**Supplemental Table 1**: ICD-10, procedural, and ATC for medications used to identify covariates’ codes

| **Disease name** | **ICD-10 code** |
| --- | --- |
| Type 2 diabetic nephropathy (DKD) | E112, E142 |
| Type 2 diabetes mellitus | E11, E12, E13, E14 |
| Type 1 diabetes mellitus | E10 |
| Hypertension | I10, I110, I120, I129, I139, I150, I151, I152, I158, I159 |
| Hyperlipidemia | E780, E781, E782, E783, E784, E785, E786, E788, E789 |
| Ischemic heart disease | I200, I201, I208, I209, I210, I211, I212, I213, I214, I219, I220, I221, I228, I229, I230, I231, I232, I233, I234, I235, I236, I238, I240, I241, I248, I249, I251, I252, I253, I254, I255, I256, I258, I259 |
| Hyperuricemia | E790 |
|  |  |
| **Procedure name** | **Procedural code** |
| Medical guidance to prevent dialysis related to diabetes | B001-27 |
| Percutaneous coronary intervention | K549, K546, K548 |
| Coronary artery bypass | K597, K598 |
| Cerebral vascular surgery | K164-3, K178 |
| Outpatient nutritional guidance | B001-00 |
| Permanent dialysis | J038, J042 |
| Kidney transplant | K780-2 |
|  |  |
| **Medication** | **ATC code** |
| Oral hypoglycemic agents | A10BA, A10BB, A10BD, A10BF, A10BG, A10BH, A10BJ, A10BK, A10BX |
| Insulin | A10AB, A10AC, A10AD, A10AE |
| Ca blocker | C08CA, C08DA, C08DB, C08EA |
| RAS antagonists | C09AA, C09CA, C09DA, C09DB, C09DX, C09XA |
| β-blocker | C07AA, C07AB, C07AG |
| Lipid-lowering agents | C10AA, C10AB, C10AC, C10AD, C10AX, C10BA, C10BX |
| Uric acid-lowering agents | M04AA, M04AB, M04AC, M04AX |

ICD-10 codes: International Classification of Diseases 10^th^ Revision diagnosis code, ATC code: Anatomical Therapeutic Chemical code, DKD: diabetic kidney disease, Ca: calcium channel, RAS: renin-angiotensin system

**Supplemental Table 2**

Baseline characteristics of the study population before and after propensity score matching for secondary outcomes: (A) death, permanent dialysis, temporary catheterization, (B) hospitalization, and (C) KDRT

| (A) | Before matching | | | After matching | | |
| --- | --- | --- | --- | --- | --- | --- |
| Variables | MDC group | Non-MDC group | SMD | MDC group | Non-MDC group | SMD |
|  | n = 1,095 | n = 9,422 |  | n = 1,090 | n = 4,904 |  |
| **Variables used for PS calculation** |  |  |  |  |  |  |
| Age (years), median (IQR) | 70 (62–76) | 70 (63–77) | 0.02 | 70 (62–76) | 70 (63–76) | 0.02 |
| Categorized, n (%) |  |  | 0.11 |  |  | 0.03 |
| 20–49 | 63 (5.8) | 559 (5.9) |  | 63 (5.8) | 284 (5.8) |  |
| 50–59 | 130 (11.9) | 1,185 (12.6) |  | 130 (11.9) | 585 (11.9) |  |
| 60–69 | 342 (31.2) | 2,889 (30.7) |  | 341 (31.3) | 1,567 (32.0) |  |
| 70–79 | 415 (37.9) | 3,220 (34.2) |  | 411 (37.7) | 1,780 (36.3) |  |
| ≥80 | 145 (13.2) | 1,569 (16.7) |  | 145 (13.3) | 688 (14.0) |  |
| Male sex, n (%) | 731 (66.8) | 6,095 (64.7) | 0.04 | 726 (66.6) | 3,241 (66.1) | 0.01 |
| Hospital size by number of beds,  n (%) |  |  | 0.36 |  |  | 0.07 |
| ≥500 | 119 (10.9) | 2,075 (22.0) |  | 119 (10.9) | 587 (12.0) |  |
| 300–499 | 667 (60.9) | 4,331 (46.0) |  | 662 (60.7) | 2,801 (57.1) |  |
| 100–299 | 309 (28.2) | 2,987 (31.7) |  | 309 (28.3) | 1,516 (30.9) |  |
| 20–99 | 0 (0.0) | 10 (0.1) |  | 0 (0.0) | 0 (0.0) |  |
| <20 | 0 (0.0) | 19 (0.2) |  | 0 (0.0) | 0 (0.0) |  |
| Duration of diabetes (years), median (IQR) | 7.2 (2.0–13.1) | 5.0 (0.6–11.0) | 0.22 | 7.1 (1.9–13.1) | 6.3 (1.1–11.8) | 0.11 |
| Categorized, n (%) |  |  | 0.21 |  |  | 0.09 |
| 0–5 | 443 (40.5) | 4,729 (50.2) |  | 443 (40.6) | 2,160 (44.0) |  |
| 5–15 | 454 (41.5) | 3,463 (36.8) |  | 453 (41.6) | 2,019 (41.2) |  |
| 15–25 | 177 (16.2) | 1100 (11.7) |  | 174 (16.0) | 648 (13.2) |  |
| ≥25 | 21 (1.9) | 130 (1.4) |  | 20 (1.8) | 77 (1.6) |  |
| Index eGFR (ml/min/1.73 m^2^), median (IQR) | 56 (42–69) | 60 (43–73) | 0.13 | 56 (42–69) | 56 (42–71) | 0.03 |
| Categorized, n (%) |  |  | 0.19 |  |  | 0.04 |
| G2: 60–90 | 456 (41.6) | 4,758 (50.5) |  | 456 (41.8) | 2,128 (43.4) |  |
| G3a: 45–60 | 320 (29.2) | 2,160 (22.9) |  | 318 (29.2) | 1,382 (28.2) |  |
| G3b: 30–45 | 201 (18.4) | 1,455 (15.4) |  | 198 (18.2) | 844 (17.2) |  |
| G4: 15–30 | 118 (10.8) | 1,049 (11.1) |  | 118 (10.8) | 550 (11.2) |  |
| Medication use, n (%) |  |  |  |  |  |  |
| Oral hypoglycemic agents | 880 (80.4) | 6,155 (65.3) | 0.34 | 875 (80.3) | 3,851 (78.5) | 0.04 |
| Insulin | 322 (29.4) | 2,279 (24.2) | 0.12 | 319 (29.3) | 1,324 (27.0) | 0.05 |
| Ca blockers | 475 (43.4) | 3,119 (33.1) | 0.21 | 472 (43.3) | 2,059 (42.0) | 0.03 |
| RAS–antagonists | 619 (56.5) | 3,923 (41.6) | 0.30 | 614 (56.3) | 2,622 (53.5) | 0.06 |
| β–blockers | 183 (16.7) | 1,196 (12.7) | 0.11 | 181 (16.6) | 741 (15.1) | 0.04 |
| Lipid–lowering agents | 535 (48.9) | 3,666 (38.9) | 0.20 | 530 (48.6) | 2,272 (46.3) | 0.05 |
| Uric acid–lowering agents | 182 (16.6) | 1,083 (11.5) | 0.15 | 178 (16.3) | 730 (14.9) | 0.04 |
| Procedure, n (%) |  |  |  |  |  |  |
| Percutaneous coronary intervention | 66 (6.0) | 216 (2.3) | 0.19 | 61 (5.6) | 186 (3.8) | 0.09 |
| Coronary artery bypass grafting | 3 (0.3) | 11 (0.1) | 0.04 | 3 (0.3) | 8 (0.2) | 0.02 |
| Cerebral vascular surgery | 1 (0.1) | 3 (0.0) | 0.02 | 1 (0.1) | 3 (0.1) | 0.01 |
|  |  |  |  |  |  |  |
| **Variables not used for PS calculation** |  |  |  |  |  |  |
| BMI (kg/m^2^), mean (SD) | 25.4 (4.4) | 24.9 (4.7) | 0.12 | 25.4 (4.4) | 25.2 (4.6) | 0.07 |
| Categorized, n (%) |  |  | 0.35 |  |  | 0.31 |
| <18.5 | 17 (1.6) | 117 (1.2) |  | 17 (1.6) | 54 (1.1) |  |
| 18.5–25 | 189 (17.3) | 1,061 (11.3) |  | 185 (17.0) | 562 (11.5) |  |
| ≥25 | 205 (18.7) | 921 (9.8) |  | 204 (18.7) | 529 (10.8) |  |
| Missing data | 684 (62.5) | 7,323 (77.7) |  | 684 (62.8) | 3,759 (76.7) |  |
| Smoking, n (%) |  |  | 0.31 |  |  | 0.28 |
| Non–smoker | 227 (20.7) | 1,125 (11.9) |  | 223 (20.5) | 614 (12.5) |  |
| Past or current smoker | 160 (14.6) | 919 (9.8) |  | 159 (14.6) | 500 (10.2) |  |
| Missing data | 708 (64.7) | 7,378 (78.3) |  | 708 (65.0) | 3,790 (77.3) |  |
| HbA1c (%), median (IQR) | 7.2 (6.6–7.9) | 7.1 (6.4–8.0) | 0.03 | 7.2 (6.6–7.9) | 7.1 (6.5–8.1) | 0.02 |
| Categorized, n (%) |  |  | 0.27 |  |  | 0.26 |
| <6.0 | 41 (3.7) | 901 (9.6) |  | 41 (3.8) | 451 (9.2) |  |
| 6.0–7.0 | 389 (35.5) | 3,349 (35.5) |  | 386 (35.4) | 1,742 (35.5) |  |
| 7.0–8.0 | 388 (35.4) | 2,582 (27.4) |  | 387 (35.5) | 1,353 (27.6) |  |
| ≥8.0 | 263 (24.0) | 2,440 (25.9) |  | 263 (24.1) | 1,292 (26.3) |  |
| Missing | 14 (1.3) | 150 (1.6) |  | 13 (1.2) | 66 (1.3) |  |
| Proteinuria^a^, n (%) |  |  | 0.38 |  |  | 0.31 |
| – | 289 (26.4) | 3,999 (42.4) |  | 289 (26.5) | 1,907 (38.9) |  |
| ± | 216 (19.7) | 1,409 (15.0) |  | 216 (19.8) | 740 (15.1) |  |
| 1+ | 201 (18.4) | 1,167 (12.4) |  | 201 (18.4) | 683 (13.9) |  |
| 2+ | 136 (12.4) | 894 (9.5) |  | 136 (12.5) | 466 (9.5) |  |
| 3+ | 81 (7.4) | 764 (8.1) |  | 81 (7.4) | 425 (8.7) |  |
| 4+ | 8 (0.7) | 140 (1.5) |  | 8 (0.7) | 74 (1.5) |  |
| Missing data | 164 (15.0) | 1,049 (11.1) |  | 159 (14.6) | 609 (12.4) |  |
| LDL–C (mg/dl), mean (SD) | 103 (32) | 108 (35) | 0.16 | 103 (32) | 107 (35) | 0.11 |
| Categorized, n (%) |  |  | 0.15 |  |  | 0.12 |
| <100 | 417 (38.1) | 3,239 (34.4) |  | 417 (38.3) | 1,758 (35.8) |  |
| 100–120 | 190 (17.4) | 1,758 (18.7) |  | 190 (17.4) | 922 (18.8) |  |
| 120–140 | 128 (11.7) | 1,272 (13.5) |  | 128 (11.7) | 617 (12.6) |  |
| 140–160 | 53 (4.8) | 660 (7.0) |  | 53 (4.9) | 334 (6.8) |  |
| ≥160 | 47 (4.3) | 513 (5.4) |  | 47 (4.3) | 246 (5.0) |  |
| Missing data | 260 (23.7) | 1,980 (21.0) |  | 255 (23.4) | 1,027 (20.9) |  |
| Uric acid (mg/dl), mean (SD) | 6.0 (1.7) | 6.2 (4.3) | 0.07 | 6.0 (1.7) | 6.2 (3.7) | 0.07 |
| Categorized, n (%) |  |  | 0.12 |  |  | 0.12 |
| <7.0 | 809 (73.9) | 6,818 (72.4) |  | 805 (73.9) | 3,533 (72.0) |  |
| 7.0–8.0 | 132 (12.1) | 940 (10.0) |  | 131 (12.0) | 499 (10.2) |  |
| 8.0–9.0 | 42 (3.8) | 371 (3.9) |  | 42 (3.9) | 192 (3.9) |  |
| ≥9.0 | 35 (3.2) | 367 (3.9) |  | 35 (3.2) | 192 (3.9) |  |
| Missing | 77 (7.0) | 926 (9.8) |  | 77 (7.1) | 488 (10.0) |  |
| Comorbidities, n (%) |  |  |  |  |  |  |
| Hypertension | 908 (82.9) | 6,874 (73.0) | 0.24 | 903 (82.8) | 3,889 (79.3) | 0.09 |
| Hyperlipidemia | 812 (74.2) | 6,045 (64.2) | 0.22 | 807 (74.0) | 3,331 (67.9) | 0.14 |
| Ischemic heart disease | 455 (41.6) | 3,329 (35.3) | 0.13 | 450 (41.3) | 1,842 (37.6) | 0.08 |
| Hyperuricemia | 232 (21.2) | 1,498 (15.9) | 0.14 | 228 (20.9) | 900 (18.4) | 0.07 |
| Observation period^b^ (years), median (IQR) | 3.5 (2.1–4.8) | 3.9 (2.5–5.8) | 0.29 | 3.5 (2.1–4.8) | 3.9 (2.5–5.9) | 0.29 |

| (B) | Before matching | | | After matching | | |
| --- | --- | --- | --- | --- | --- | --- |
| Variables | MDC group | Non-MDC group | SMD | MDC group | Non-MDC group | SMD |
|  | n = 845 | n = 6,618 |  | n = 840 | n = 3,541 |  |
| **Variables used for PS calculation** |  |  |  |  |  |  |
| Age (years), median (IQR) | 69 (62–75) | 69 (62–76) | 0.01 | 69 (62–75) | 69 (62–76) | 0.01 |
| Categorized, n (%) |  |  | 0.13 |  |  | 0.05 |
| 20–49 | 47 (5.6) | 421 (6.4) |  | 47 (5.6) | 220 (6.2) |  |
| 50–59 | 104 (12.3) | 900 (13.6) |  | 104 (12.4) | 422 (11.9) |  |
| 60–69 | 277 (32.8) | 2,136 (32.3) |  | 276 (32.9) | 1,193 (33.7) |  |
| 70–79 | 320 (37.9) | 2,190 (33.1) |  | 316 (37.6) | 1,264 (35.7) |  |
| ≥80 | 97 (11.5) | 971 (14.7) |  | 97 (11.5) | 442 (12.5) |  |
| Male sex, n (%) | 293 (34.7) | 2,415 (36.5) | 0.04 | 292 (34.8) | 1,254 (35.4) | 0.01 |
| Hospital size by number of beds,  n (%) |  |  | 0.31 |  |  | 0.13 |
| ≥500 | 91 (10.8) | 1,221 (18.4) |  | 91 (10.8) | 429 (12.1) |  |
| 300–499 | 500 (59.2) | 2,993 (45.2) |  | 495 (58.9) | 1,865 (52.7) |  |
| 100–299 | 254 (30.1) | 2,381 (36.0) |  | 254 (30.2) | 1,247 (35.2) |  |
| 20–99 | 0 (0.0) | 7 (0.1) |  |  |  |  |
| <20 | 0 (0.0) | 16 (0.2) |  |  |  |  |
| Duration of diabetes (years), median (IQR) | 7.1 (2.1– 13.0) | 5.4 (1.1– 11.0) | 0.19 | 7.1 (2.0– 12.9) | 6.3 (1.4– 11.7) | 0.10 |
| Categorized, (years), n (%) |  |  | 0.18 |  |  | 0.09 |
| 0–5 | 335 (39.6) | 3,173 (47.9) |  | 335 (39.9) | 1,544 (43.6) |  |
| 5–15 | 362 (42.8) | 2,571 (38.8) |  | 361 (43.0) | 1,484 (41.9) |  |
| 15–25 | 133 (15.7) | 790 (11.9) |  | 130 (15.5) | 462 (13.0) |  |
| ≥25 | 15 (1.8) | 84 (1.3) |  | 14 (1.7) | 51 (1.4) |  |
| Index eGFR (ml/min/1.73m^2^), median (IQR) | 57 (44– 71) | 63 (49– 75) | 0.23 | 58 (44– 71) | 59 (46– 73) | 0.09 |
| Categorized, n (%) |  |  | 0.25 |  |  | 0.08 |
| G2: 60–90 | 375 (44.4) | 3,764 (56.9) |  | 375 (44.6) | 1,697 (47.9) |  |
| G3a: 45–60 | 250 (29.6) | 1,548 (23.4) |  | 247 (29.4) | 1,036 (29.3) |  |
| G3b: 30–45 | 142 (16.8) | 812 (12.3) |  | 140 (16.7) | 524 (14.8) |  |
| G4: 15–30 | 78 (9.2) | 494 (7.5) |  | 78 (9.3) | 284 (8.0) |  |
| Medication use, n (%) |  |  |  |  |  |  |
| Oral hypoglycemic agents | 669 (79.2) | 4,094 (61.9) | 0.39 | 664 (79.0) | 2,716 (76.7) | 0.06 |
| Insulin | 210 (24.9) | 1,078 (16.3) | 0.21 | 207 (24.6) | 716 (20.2) | 0.11 |
| Ca blockers | 351 (41.5) | 1,849 (27.9) | 0.29 | 347 (41.3) | 1,353 (38.2) | 0.06 |
| RAS–antagonists | 460 (54.4) | 2,456 (37.1) | 0.35 | 455 (54.2) | 1,792 (50.6) | 0.07 |
| β–blockers | 130 (15.4) | 575 (8.7) | 0.21 | 127 (15.1) | 425 (12.0) | 0.09 |
| Lipid–lowering agents | 391 (46.3) | 2,402 (36.3) | 0.20 | 386 (46.0) | 1,591 (44.9) | 0.02 |
| Uric acid–lowering agents | 126 (14.9) | 554 (8.4) | 0.21 | 123 (14.6) | 433 (12.2) | 0.07 |
| Procedure, n (%) |  |  |  |  |  |  |
| Percutaneous coronary intervention | 38 (4.5) | 97 (1.5) | 0.18 | 33 (3.9) | 80 (2.3) | 0.10 |
| Coronary artery bypass grafting | 3 (0.4) | 6 (0.1) | 0.06 | 3 (0.4) | 4 (0.1) | 0.05 |
| Cerebral vascular surgery | 0 (0.0) | 1 (0.0) | 0.02 | 0 (0.0) | 0 (0.0) | <0.001 |
|  |  |  |  |  |  |  |
| **Variables not used for PS calculation** |  |  |  |  |  |  |
| BMI (kg/m^2^), mean (SD) | 25.4 (4.3) | 25.0 (4.8) | 0.10 | 25.4 (4.3) | 25.3 (4.8) | 0.04 |
| Categorized, n (%) |  |  | 0.45 |  |  | 0.41 |
| <18.5 | 9 (1.1) | 58 (0.9) |  | 9 (1.1) | 25 (0.7) |  |
| 18.5–25 | 126 (14.9) | 462 (7.0) |  | 123 (14.6) | 261 (7.4) |  |
| ≥25 | 138 (16.3) | 413 (6.2) |  | 136 (16.2) | 249 (7.0) |  |
| Missing data | 572 (67.7) | 5,685 (85.9) |  | 572 (68.1) | 3006 (84.9) |  |
| Smoking, n (%) |  |  | 0.40 |  |  | 0.37 |
| Non–smoker | 149 (17.6) | 504 (7.6) |  | 145 (17.3) | 278 (7.9) |  |
| Past or current smoker | 103 (12.2) | 393 (5.9) |  | 102 (12.1) | 232 (6.6) |  |
| Missing data | 593 (70.2) | 5,721 (86.4) |  | 593 (70.6) | 3,031 (85.6) |  |
| HbA1c (%), median (IQR) | 7.1 (6.6– 7.8) | 7.0 (6.4– 7.8) | 0.03 | 7.1 (6.6– 7.8) | 7.0 (6.4– 7.8) | 0.03 |
| Categorized, n (%) |  |  | 0.25 |  |  | 0.26 |
| <6.0 | 31 (3.7) | 609 (9.2) |  | 31 (3.7) | 330 (9.3) |  |
| 6.0–7.0 | 312 (36.9) | 2,563 (38.7) |  | 310 (36.9) | 1,375 (38.8) |  |
| 7.0–8.0 | 312 (36.9) | 1,977 (29.9) |  | 311 (37.0) | 1,054 (29.8) |  |
| ≥8.0 | 178 (21.1) | 1,376 (20.8) |  | 177 (21.1) | 743 (21.0) |  |
| Missing data | 12 (1.4) | 93 (1.4) |  | 11 (1.3) | 39 (1.1) |  |
| Proteinuria^a^, n (%) |  |  | 0.44 |  |  | 0.36 |
| – | 234 (27.7) | 3,169 (47.9) |  | 234 (27.9) | 1,545 (43.6) |  |
| ± | 180 (21.3) | 1,028 (15.5) |  | 180 (21.4) | 555 (15.7) |  |
| 1+ | 150 (17.8) | 757 (11.4) |  | 150 (17.9) | 453 (12.8) |  |
| 2+ | 90 (10.7) | 514 (7.8) |  | 90 (10.7) | 279 (7.9) |  |
| 3+ | 59 (7.0) | 398 (6.0) |  | 59 (7.0) | 237 (6.7) |  |
| 4+ | 4 (0.5) | 70 (1.1) |  | 4 (0.5) | 45 (1.3) |  |
| Missing data | 128 (15.1) | 682 (10.3) |  | 123 (14.6) | 427 (12.1) |  |
| LDL–C (mg/dl), mean (SD) | 104 (31) | 109 (32) | 0.15 | 104 (31) | 107 (32) | 0.09 |
| Categorized, n (%) |  |  | 0.17 |  |  | 0.12 |
| <100 | 300 (35.5) | 2,214 (33.5) |  | 300 (35.7) | 1,269 (35.8) |  |
| 100–120 | 151 (17.9) | 1,318 (19.9) |  | 151 (18.0) | 677 (19.1) |  |
| 120–140 | 109 (12.9) | 925 (14.0) |  | 109 (13.0) | 457 (12.9) |  |
| 140–160 | 43 (5.1) | 493 (7.4) |  | 43 (5.1) | 244 (6.9) |  |
| ≥160 | 29 (3.4) | 312 (4.7) |  | 29 (3.5) | 149 (4.2) |  |
| Missing | 213 (25.2) | 1,356 (20.5) |  | 208 (24.8) | 745 (21.0) |  |
| Uric acid (mg/dl), mean (SD) | 5.9 (1.5) | 6.0 (3.9) | 0.04 | 5.9 (1.5) | 6.1 (3.5) | 0.07 |
| Categorized, n (%) |  |  | 0.15 |  |  | 0.16 |
| <7.0 | 632 (74.8) | 4,856 (73.4) |  | 628 (74.8) | 2,533 (71.5) |  |
| 7.0–8.0 | 96 (11.4) | 599 (9.1) |  | 95 (11.3) | 339 (9.6) |  |
| 8.0–9.0 | 30 (3.6) | 203 (3.1) |  | 30 (3.6) | 121 (3.4) |  |
| ≥9.0 | 19 (2.2) | 175 (2.6) |  | 19 (2.3) | 112 (3.2) |  |
| Missing data | 68 (8.0) | 785 (11.9) |  | 68 (8.1) | 436 (12.3) |  |
| Comorbidities, n (%) |  |  |  |  |  |  |
| Hypertension | 690 (81.7) | 4,765 (72.0) | 0.23 | 685 (81.5) | 2,787 (78.7) | 0.07 |
| Hyperlipidemia | 609 (72.1) | 4,352 (65.8) | 0.14 | 604 (71.9) | 2,479 (70.0) | 0.04 |
| Ischemic heart disease | 330 (39.1) | 2,149 (32.5) | 0.14 | 325 (38.7) | 1,228 (34.7) | 0.08 |
| Hyperuricemia | 173 (20.5) | 912 (13.8) | 0.18 | 170 (20.2) | 597 (16.9) | 0.09 |
| Observation period^b^ (years), median (IQR) | 3.6 (2.3–4.9) | 4.3 (2.7–6.2) | 0.30 | 3.6 (2.3–4.9) | 4.3 (2.7–6.2) | 0.38 |

| (C) | Before matching | | | After matching | | |
| --- | --- | --- | --- | --- | --- | --- |
| Variables | MDC group | Non–MDC group | SMD | MDC group | Non-MDC group | SMD |
|  | n = 1065 | n = 9022 |  | n = 1062 | n = 4695 |  |
| **Variables used for PS calculation** |  |  |  |  |  |  |
| Age (years), median (IQR) | 70 (63–76) | 70 (63–77) | 0.02 | 70 (62–76) | 70 (63–76) | 0.02 |
| Categorized, n (%) |  |  | 0.11 |  |  | 0.03 |
| 20–49 | 59 (5.5) | 534 (5.9) |  | 59 (5.6) | 266 (5.7) |  |
| 50–59 | 127 (11.9) | 1,122 (12.4) |  | 127 (12.0) | 547 (11.7) |  |
| 60–69 | 330 (31.0) | 2,774 (30.7) |  | 330 (31.1) | 1,448 (30.8) |  |
| 70–79 | 410 (38.5) | 3,109 (34.5) |  | 407 (38.3) | 1,781 (37.9) |  |
| ≥80 | 139 (13.1) | 1,483 (16.4) |  | 139 (13.1) | 653 (13.9) |  |
| Male sex, n (%) | 357 (33.5) | 3,197 (35.4) | 0.04 | 357 (33.6) | 1,609 (34.3) | 0.01 |
| Hospital size by number of beds,  n (%) |  |  | 0.37 |  |  | 0.08 |
| ≥500 | 110 (10.3) | 1,906 (21.1) |  | 110 (10.4) | 516 (11.0) |  |
| 300–499 | 654 (61.4) | 4,166 (46.2) |  | 301 (28.3) | 1,485 (31.6) |  |
| 100–299 | 301 (28.3) | 2,922 (32.4) |  | 651 (61.3) | 2,694 (57.4) |  |
| 20–99 | 0 (0.0) | 10 (0.1) |  |  |  |  |
| <20 | 0 (0.0) | 18 (0.2) |  |  |  |  |
| Duration of diabetes (years), median (IQR) | 7.2 (2.0–13.1) | 5.0 (0.7–11.0) | 0.22 | 7.2 (2.0–13.1) | 6.3 (1.1–11.9) | 0.11 |
| Categorized, n (%) |  |  | 0.20 |  |  | 0.09 |
| 0–5 | 430 (40.4) | 4,485 (49.7) |  | 430 (40.5) | 2,085 (44.4) |  |
| 5–15 | 442 (41.5) | 3,358 (37.2) |  | 442 (41.6) | 1,897 (40.4) |  |
| 15–25 | 173 (16.2) | 1,056 (11.7) |  | 171 (16.1) | 639 (13.6) |  |
| ≥25 | 20 (1.9) | 123 (1.4) |  | 19 (1.8) | 74 (1.6) |  |
| Index eGFR (ml/min/1.73 m^2^), median (IQR) | 57 (43–70) | 61 (46–74) | 0.17 | 57 (43–70) | 57 (44–71) | 0.04 |
| Categorized, n (%) |  |  | 0.20 |  |  | 0.03 |
| G2: 60–90 | 456 (42.8) | 4,756 (52.7) |  | 456 (42.9) | 2,074 (44.2) |  |
| G3a: 45–60 | 320 (30.0) | 2,148 (23.8) |  | 318 (29.9) | 1,390 (29.6) |  |
| G3b: 30–45 | 200 (18.8) | 1,413 (15.7) |  | 199 (18.7) | 842 (17.9) |  |
| G4: 15–30 | 89 (8.4) | 705 (7.8) |  | 89 (8.4) | 389 (8.3) |  |
| Medication use, n (%) |  |  |  |  |  |  |
| Oral hypoglycemic agents | 858 (80.6) | 5,922 (65.6) | 0.34 | 855 (80.5) | 3,651 (77.8) | 0.07 |
| Insulin | 308 (28.9) | 2,141 (23.7) | 0.12 | 306 (28.8) | 1,242 (26.5) | 0.05 |
| Ca blockers | 460 (43.2) | 2,877 (31.9) | 0.24 | 458 (43.1) | 1,862 (39.7) | 0.07 |
| RAS–antagonists | 599 (56.2) | 3,695 (41.0) | 0.31 | 596 (56.1) | 2,477 (52.8) | 0.07 |
| β–blockers | 177 (16.6) | 1,097 (12.2) | 0.13 | 176 (16.6) | 698 (14.9) | 0.05 |
| Lipid–lowering agents | 522 (49.0) | 3,486 (38.6) | 0.21 | 520 (49.0) | 2,195 (46.8) | 0.04 |
| Uric acid–lowering agents | 172 (16.2) | 959 (10.6) | 0.16 | 170 (16.0) | 662 (14.1) | 0.05 |
| Procedure, n (%) |  |  |  |  |  |  |
| Percutaneous coronary intervention | 61 (5.7) | 206 (2.3) | 0.18 | 58 (5.5) | 173 (3.7) | 0.09 |
| Coronary artery bypass grafting | 3 (0.3) | 10 (0.1) | 0.04 | 3 (0.3) | 8 (0.2) | 0.02 |
| Cerebral vascular surgery | 1 (0.1) | 3 (0.0) | 0.02 | 1 (0.1) | 3 (0.1) | 0.01 |
|  |  |  |  |  |  |  |
| **Variables not used for PS calculation** |  |  |  |  |  |  |
| BMI (kg/m^2^), mean (SD) | 25.5 (4.4) | 24.9 (4.7) | 0.12 | 25.5 (4.4) | 25.1 (4.7) | 0.09 |
| Categorized, n (%) |  |  | 0.35 |  |  | 0.32 |
| <18.5 | 16 (1.5) | 115 (1.3) |  | 16 (1.5) | 56 (1.2) |  |
| 18.5–25 | 181 (17.0) | 983 (10.9) |  | 180 (16.9) | 521 (11.1) |  |
| ≥25 | 199 (18.7) | 870 (9.6) |  | 198 (18.6) | 500 (10.6) |  |
| Missing data | 669 (62.8) | 7,054 (78.2) |  | 668 (62.9) | 3,618 (77.1) |  |
| Smoking, n (%) |  |  | 0.31 |  |  | 0.28 |
| Non–smoker | 219 (20.6) | 1,052 (11.7) |  | 217 (20.4) | 564 (12.0) |  |
| Past or current smoker | 154 (14.5) | 868 (9.6) |  | 154 (14.5) | 489 (10.4) |  |
| Missing data | 692 (65.0) | 7,102 (78.7) |  | 691 (65.1) | 3,642 (77.6) |  |
| HbA1c (%), median (IQR) | 7.2 (6.6–7.9) | 7.1 (6.5–8.1) | 0.03 | 7.2 (6.6–7.9) | 7.1 (6.4–8.0) | 0.01 |
| Categorized, n (%) |  |  | 0.27 |  |  | 0.27 |
| <6.0 | 36 (3.4) | 797 (8.8) |  | 36 (3.4) | 434 (9.2) |  |
| 6.0–7.0 | 379 (35.6) | 3,208 (35.6) |  | 377 (35.5) | 1,673 (35.6) |  |
| 7.0–8.0 | 380 (35.7) | 2,519 (27.9) |  | 380 (35.8) | 1,324 (28.2) |  |
| ≥8.0 | 258 (24.2) | 2,368 (26.2) |  | 258 (24.3) | 1,204 (25.6) |  |
| Missing data | 12 (1.1) | 130 (1.4) |  | 11 (1.0) | 60 (1.3) |  |
| Proteinuria^a^, n (%) |  |  | 0.38 |  |  | 0.30 |
| – | 288 (27.0) | 3,975 (44.1) |  | 288 (27.1) | 1,880 (40.0) |  |
| ± | 216 (20.3) | 1,393 (15.4) |  | 216 (20.3) | 726 (15.5) |  |
| 1+ | 199 (18.7) | 1,134 (12.6) |  | 198 (18.6) | 655 (14.0) |  |
| 2+ | 130 (12.2) | 823 (9.1) |  | 130 (12.2) | 454 (9.7) |  |
| 3+ | 68 (6.4) | 595 (6.6) |  | 68 (6.4) | 331 (7.1) |  |
| 4+ | 6 (0.6) | 99 (1.1) |  | 6 (0.6) | 53 (1.1) |  |
| Missing data | 158 (14.8) | 1,003 (11.1) |  | 156 (14.7) | 596 (12.7) |  |
| LDL–C (mg/dl), mean (SD) | 103 (32) | 108 (34) | 0.15 | 103 (32) | 106 (34) | 0.08 |
| Categorized, n (%) |  |  | 0.14 |  |  | 0.09 |
| <100 | 404 (37.9) | 3,102 (34.4) |  | 404 (38.0) | 1,711 (36.4) |  |
| 100–120 | 184 (17.3) | 1,707 (18.9) |  | 184 (17.3) | 907 (19.3) |  |
| 120–140 | 125 (11.7) | 1,230 (13.6) |  | 124 (11.7) | 591 (12.6) |  |
| 140–160 | 52 (4.9) | 629 (7.0) |  | 52 (4.9) | 278 (5.9) |  |
| ≥160 | 46 (4.3) | 468 (5.2) |  | 46 (4.3) | 217 (4.6) |  |
| Missing data | 254 (23.8) | 1,886 (20.9) |  | 252 (23.7) | 991 (21.1) |  |
| Uric acid (mg/dl), mean (SD) | 5.9 (1.7) | 6.1 (4.2) | 0.05 | 5.9 (1.7) | 6.2 (4.3) | 0.08 |
| Categorized, n (%) |  |  | 0.12 |  |  | 0.11 |
| <7.0 | 793 (74.5) | 6,604 (73.2) |  | 790 (74.4) | 3,405 (72.5) |  |
| 7.0–8.0 | 126 (11.8) | 878 (9.7) |  | 126 (11.9) | 475 (10.1) |  |
| 8.0–9.0 | 38 (3.6) | 326 (3.6) |  | 38 (3.6) | 182 (3.9) |  |
| ≥9.0 | 31 (2.9) | 299 (3.3) |  | 31 (2.9) | 170 (3.6) |  |
| Missing data | 77 (7.2) | 915 (10.1) |  | 77 (7.3) | 463 (9.9) |  |
| Comorbidities, n (%) |  |  |  |  |  |  |
| Hypertension | 882 (82.8) | 6,520 (72.3) | 0.26 | 879 (82.8) | 3,687 (78.5) | 0.11 |
| Hyperlipidemia | 794 (74.6) | 5,804 (64.3) | 0.22 | 792 (74.6) | 3,216 (68.5) | 0.14 |
| Ischemic heart disease | 441 (41.4) | 3,159 (35.0) | 0.13 | 438 (41.2) | 1,745 (37.2) | 0.08 |
| Hyperuricemia | 220 (20.7) | 1,364 (15.1) | 0.15 | 218 (20.5) | 838 (17.8) | 0.07 |
| Observation period^b^ (years), median (IQR) | 3.5 (2.1–4.9) | 4.0 (2.5–5.9) | 0.29 | 3.5 (2.1–4.9) | 3.9 (2.5–5.9) | 0.29 |

^a^ Urine dipstick evaluation

^b^ From the index date to the date of the last observation in the database

PS: propensity score, MDC: multidisciplinary care, IQR: interquartile range, eGFR: estimated glomerular filtration rate, Ca: calcium channel, RAS: renin-angiotensin system, BMI: body mass index, SD: standard deviation, HbA1c: glycated hemoglobin, LDL-C: low-density lipoprotein cholesterol, SMD: standardized mean difference, KFRT: kidney failure with replacement therapy

**Supplemental Table 3**

Frequency of events and hazard ratios (HRs) for secondary outcomes in the sensitivity analyses compared with the main analysis

| Outcome: **Death** | Events | Patients | Person-days | Incidence rate^a^  (95% CI) | Adjusted HR  (95% CI) |
| --- | --- | --- | --- | --- | --- |
| **Changing the definition of censoring** |  |  |  |  |  |
| Non-MDC group | 342 | 4,904 | 5,630,780 | 0.61 (0.54–0.68) | Ref |
| MDC group | 55 | 1,090 | 1,084,772 | 0.51 (0.38–0.66) | 0.87 (0.65–1.15) |
| **Changing the definition of the MDC and non-MDC groups** |  |  |  |  |  |
| Non-MDC group | 236 | 3,515 | 4,258,874 | 0.55 (0.49–0.63) | Ref |
| MDC group | 37 | 719 | 697,461 | 0.53 (0.38–0.73) | 0.96 (0.68–1.36) |
| **Setting an upper limit for the observation period of 3 years** |  |  |  |  |  |
| Non-MDC group | 236 | 4,904 | 4,006,099 | 0.59 (0.52–0.67) | Ref |
| MDC group | 32 | 1,090 | 827,236 | 0.39 (0.26–0.55) | 0.68 (0.47–0.98) |
| Outcome: **Permanent dialysis** | Events | Patients | Person-days | Incidence rate^a^ (95% CI) | Adjusted HR (95% CI) |
| **Changing the definition of censoring** |  |  |  |  |  |
| Non-MDC group | 219 | 4,904 | 5,481,769 | 0.40 (0.35–0.46) | Ref |
| MDC group | 34 | 1,090 | 1,063,035 | 0.32 (0.22–0.45) | 0.85 (0.59–1.22) |
| **Changing the definition of the MDC and non-MDC groups** |  |  |  |  |  |
| Non-MDC group | 152 | 3,515 | 4,152,292 | 0.37 (0.31–0.43) | Ref |
| MDC group | 26 | 719 | 680,488 | 0.38 (0.25–0.56) | 1.22 (0.80–1.86) |
| **Setting an upper limit for the observation period of 3 years** |  |  |  |  |  |
| Non-MDC group | 160 | 4,904 | 3,937,782 | 0.41 (0.35–0.47) | Ref |
| MDC group | 26 | 1,090 | 815,534 | 0.32 (0.21–0.47) | 0.82 (0.54–1.25) |
| Outcome: **Hospitalization** | Events | Patients | Person-days | Incidence rate^a^ (95% CI) | Adjusted HR (95% CI) |
| **Changing the definition of censoring** |  |  |  |  |  |
| Non-MDC group | 1,350 | 3,541 | 3,403,530 | 3.97 (3.76–4.18) | Ref |
| MDC group | 302 | 840 | 667,727 | 4.52 (4.03–5.06) | 1.10 (0.97–1.24) |
| **Changing the definition of the MDC and non-MDC groups** |  |  |  |  |  |
| Non-MDC group | 1,127 | 2,694 | 2,621,944 | 4.30 (4.05–4.56) | Ref |
| MDC group | 203 | 572 | 444,173 | 4.57 (3.96–5.24) | 1.03 (0.89–1.20) |
| **Setting an upper limit for the observation period of 3 years** |  |  |  |  |  |
| Non-MDC group | 1,022 | 3,541 | 2,579,207 | 3.96 (3.72–4.21) | Ref |
| MDC group | 252 | 840 | 536,648 | 4.70 (4.13–5.31) | 1.14 (0.99–1.31) |
| Outcome: **Temporary catheterization** | Events | Patients | Person-days | Incidence rate^a^ (95% CI) | Adjusted HR (95% CI) |
| **Changing the definition of censoring** |  |  |  |  |  |
| Non-MDC group | 10 | 4,904 | 5,623,627 | 0.018 (0.009–0.033) | Ref |
| MDC group | 2 | 1,090 | 1,084,558 | 0.018 (0.002–0.067) | 1.07 (0.23–4.99) |
| **Changing the definition of the MDC and non-MDC groups** |  |  |  |  |  |
| Non-MDC group | 10 | 3,515 | 4,253,848 | 0.024 (0.011–0.043) | Ref |
| MDC group | 1 | 719 | 697,313 | 0.014 (0.0004–0.08) | 0.60 (0.07–4.91) |
| **Setting an upper limit for the observation period of 3 years** |  |  |  |  |  |
| Non-MDC group | 7 | 4,904 | 4,001,437 | 0.017 (0.007–0.036) | Ref |
| MDC group | 1 | 1,090 | 827,128 | 0.012 (0.003–0.067) | 0.67 (0.08–5.63) |
| Outcome: **KFRT** | Events | Patients | Person-days | Incidence rate^a^ (95% CI) | Adjusted HR (95% CI) |
| **Changing the definition of censoring** |  |  |  |  |  |
| Non-MDC group | 285 | 4,695 | 5,200,237 | 0.55 (0.49–0.62) | Ref |
| MDC group | 65 | 1,062 | 1,023,052 | 0.64 (0.49–0.81) | 1.14 (0.87–1.49) |
| **Changing the definition of the MDC and non-MDC groups** |  |  |  |  |  |
| Non-MDC group | 254 | 3,420 | 5,444,444 | 0.47 (0.41–0.53) | Ref |
| MDC group | 39 | 703 | 940,311 | 0.41 (0.29–0.57) | 1.19 (0.85–1.69) |
| **Setting an upper limit for the observation period of 3 years** |  |  |  |  |  |
| Non-MDC group | 245 | 4,695 | 3,737,749 | 0.66 (0.58–0.74) | Ref |
| MDC group | 47 | 1,062 | 727,752 | 0.65 (0.47–0.86) | 0.92 (0.67–1.25) |

The adjusted models included adjustments for sex, age (categorized), number of hospital beds, eGFR(categorized), duration of diabetes (categorized), medication use, and procedures.

^a^ Incidence rate per 10,000 person-days

MDC: multidisciplinary care, HR: hazard ratio, CI: confidence interval, eGFR: estimated glomerular filtration rate KFRT: kidney failure with replacement therapy

**Supplemental Table 4**

Comparison of the number of eGFR measurements between the MDC and non-MDC groups for the primary outcome

|  | MDC group  n = 1,039 | Non-MDC group  n = 4,575 | SMD |
| --- | --- | --- | --- |
| Total number of eGFR measurements within the observation period^a^, median (IQR) | 22 (13–33) | 22 (13–36) | 0.07 |
| Observation period^a^, (years), median (IQR) | 3.5 (2.1–4.9) | 3.9 (2.6–5.9) | 0.30 |
| Average number of eGFR measurements per year, median (IQR) | 6.5 (5.0–8.5) | 5.9 (4.0– 8.3) | 0.09 |

^a^ From the index date to the date of occurrence of the primary outcome, death, or the last observation in the database

eGFR: estimated glomerular filtration rate, MDC: multidisciplinary care, SMD: standardized mean difference, IQR: interquartile range

**Supplemental Table 5**

Evaluation of outpatient nutritional guidance for all patients in the RWD database

|  | Patients in the RWD database  n = 484,635, (%) | | SMD |
| --- | --- | --- | --- |
|  | MDC group^a^  n = 328,580 | Non-MDC group^b^  n = 156,055 |  |
| Outpatient nutritional guidance | 328,580 (100.0) | 155,545 (99.7) | 0.08 |

For all patients in the RWD database, we assessed whether the fee for outpatient nutritional guidance was assigned.

^a^ The fee for medical guidance to prevent dialysis in patients with diabetes was assigned at least once within the observation period in the database.

^b^ The fee for medical guidance to prevent dialysis in patients with diabetes was not calculated within the observation period in the database.

**Supplemental Figure 1**

The time window for this study

Cohort entry

(T2DN diagnosis)

Day 0 (index date)

INCL1

(T2DM diagnosis)

Days [-∞, +0]

EXCL1

(T1DM diagnosis)

Days [-∞, +0]

EXCL3^c^

(MDC data)

Days [-∞, -1]

INCL2

(Index eGFR)

Days [-90, +90] ^a^

EXCL2^b^

(KRT data, 40% eGFR decline)

Days [-∞, +365], [+1, +365]

INCL3

(Age ≥20)

Days [-0, +0]

COV1

(Comorbidities, procedure, smoking, BMI)

Days [-∞, +0]

COV2

(Age, sex, hospital bed)

Days [-0, +0]

COV3

(Baseline laboratory data)

Days [-90, +90] ^a^

COV4

(Drug data)

Days [-30, +30]

EXPO

(MDC data)

Days [+0, +365]

Follow up window

Days [+366, Censor ^d^]

Time

^a^ Data closest to the index date in the case of multiple datapoints

^b^ Wash-out window for outcome

^c^ Washout window for exposure

^d^ Death or date of the last observation in the database

INCL: inclusion assessment window, EXCL: exclusion assessment window, EXPO: exposure assessment window, COV: covariate assessment window, T2DN: type 2 diabetic nephropathy, T2DM: type 2 diabetes mellitus, T1DM: type 1 diabetes mellitus, KRT: kidney replacement therapy, eGFR:estimated glomerular filtration rate, MDC: multidisciplinary care, BMI: body mass index

**Supplemental Figure 2**

Distribution of propensity scores before and after matching


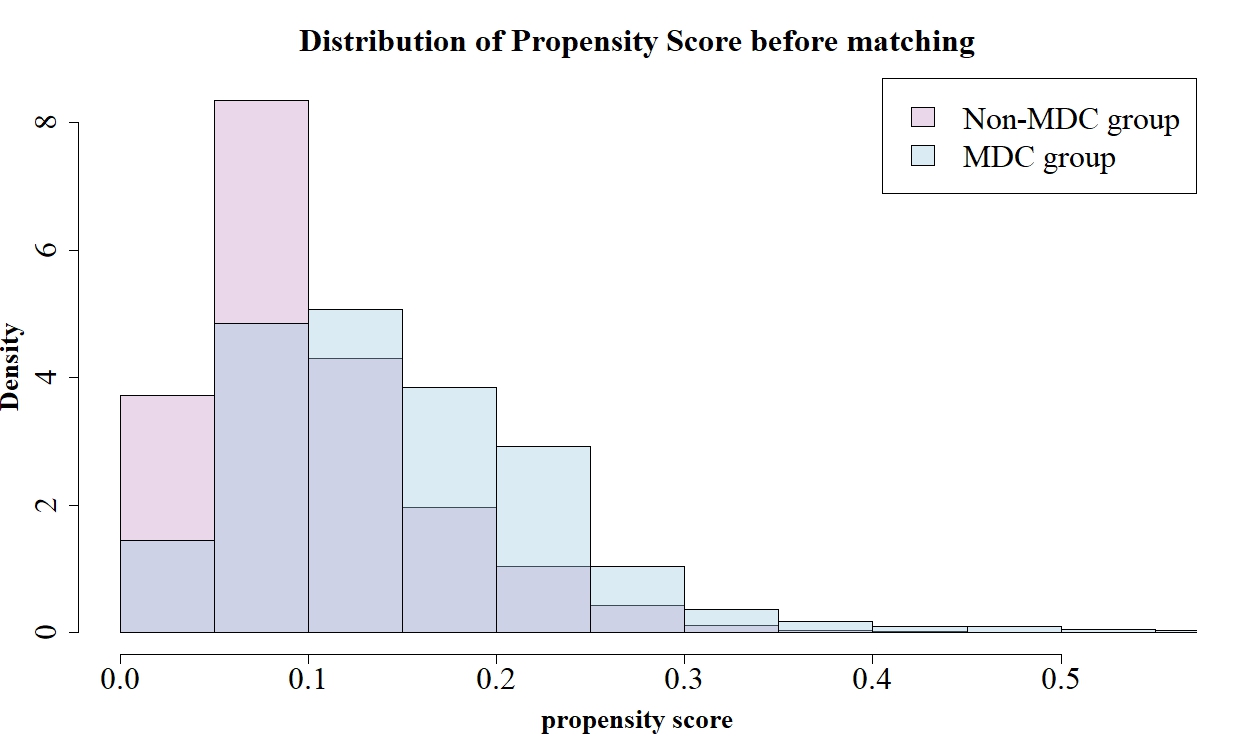


**
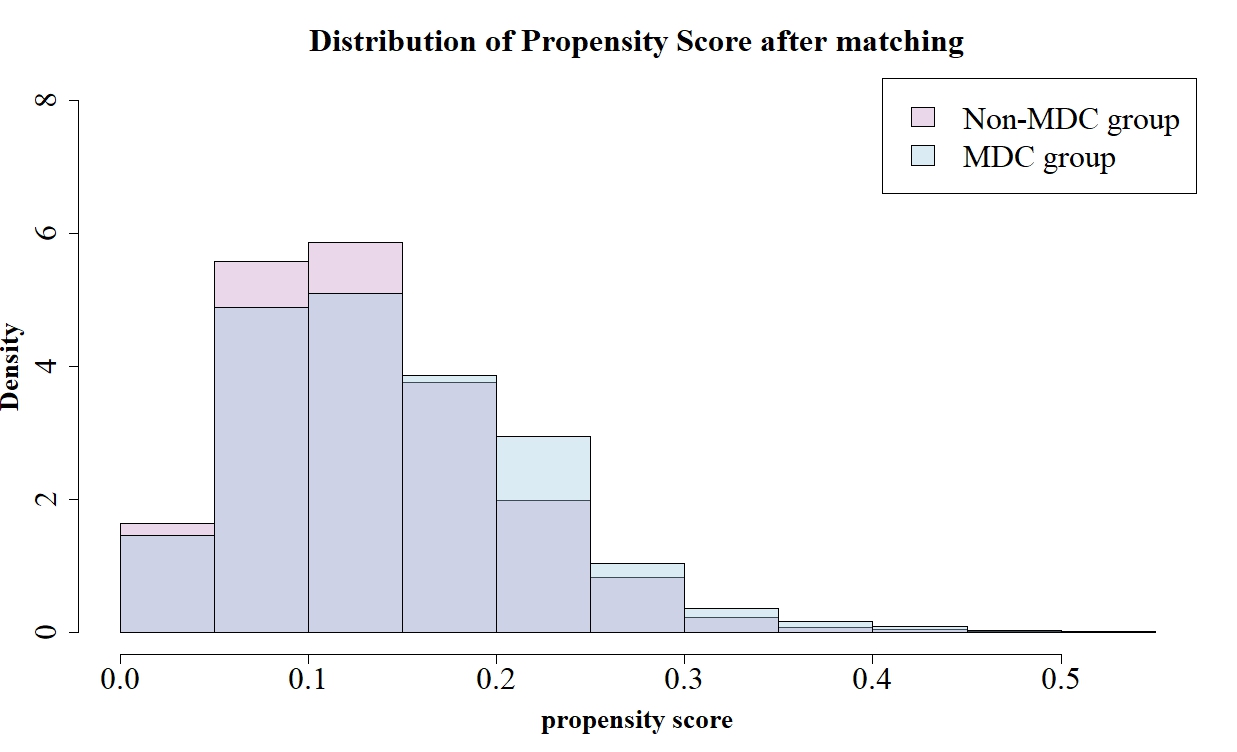
**

MDC: multidisciplinary care

**Supplemental Figure 3**

Covariate balance in propensity score matching

**
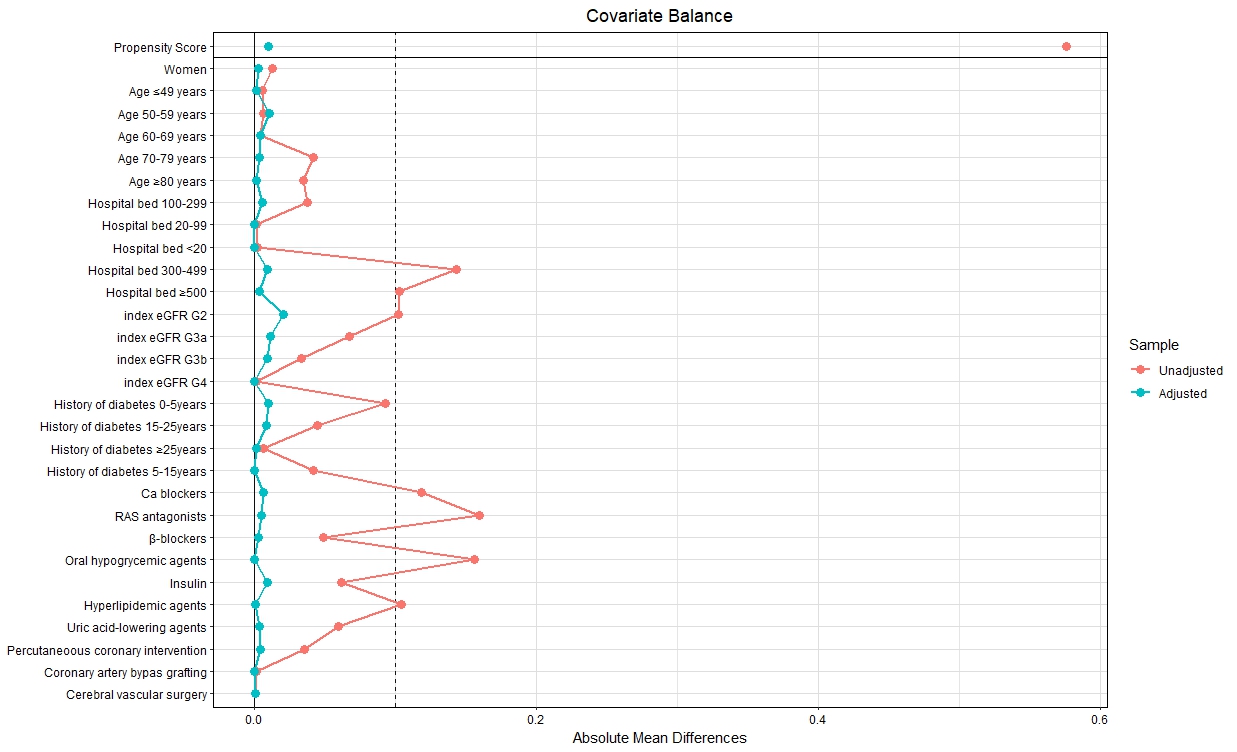
**

eGFR: estimated glomerular filtration rate, Ca: calcium channel, RAS, renin-angiotensin system

MDC group

n = 1,090

Non-MDC group

n = 4,904

**1:5**

**Propensity score matching**

Unmatched n = 4,523

Non-MDC group before matching

n = 9,422

MDC group before matching

n = 1,095

Fee for guidance within 1 year of the index date

(MDC interval ≤365 days^a^)

n = 1,095

Fee for guidance

after 1 year from the index date

(MDC interval >365 days)

n = 446

No fee for guidance within observation period

n = 8,976

Eligible patients

n = 10,517

Patients with diabetes prescriptions in the RWD database

n = 484,635

T2DKD diagnosis

n = 66,316

Excluded n = 418,319

No T2DKD diagnosis

**Supplemental Figure 4**

Flow diagrams for the secondary outcomes: (A) death, permanent dialysis, temporary catheterization, (B) hospitalization, and (C) KFRT

Excluded (n = 55,799)

Under 19 years old (n = 153)

Index eGFR <15 or >90 mL/min/1.73 m^2^ or no eGFR data

(n = 42,395)

Diagnosis history of T1DM (n = 3,170)

History of KRT before or within 1 year of the index date (n = 309)

Follow-up observation period less than 1 year or study period before April 2012 (n = 8,127)

No T2DM diagnosis before the index date (n = 1,573)

History of MDC before the index date (n = 72)

(A)

MDC group

n = 840

Non-MDC group

n = 3,541

**1:5**

**Propensity score matching**

Unmatched

n = 3,082

Non-MDC group before matching

n = 6,618

MDC group before matching

n = 845

Fee for guidance within 1 year of the index date

(MDC interval ≤365 days^a^)

n = 845

Fee for guidance

after 1 year from the index date

(MDC interval >365 days)

n = 352

No fee for guidance within observation period

n = 6,266

Eligible patients

n = 7,463

Patients with diabetes prescriptions in the RWD database

n = 484,635

T2DKD diagnosis

n = 66,316

Excluded n = 418,319

No T2DKD diagnosis

(B)

Excluded (n = 58,853)

Under 19 years old (n = 153)

Index eGFR <15 or >90 mL/min/1.73 m^2^ or no eGFR data

(n = 42,395)

Diagnosis history of T1DM (n = 3170)

History of KRT before or within 1 year of the index date (n = 309)

Follow-up observation period less than 1 year or study period before April 2012 (n = 8,127)

No T2DM diagnosis before the index date (n = 1,573)

History of MDC before the index date (n = 72)

Hospitalization occurred within the landmark time (n=3054)

MDC group

n = 1,062

Non-MDC group

n = 4,695

**1:5**

**Propensity score matching**

Unmatched

n = 4,330

Non-MDC group before matching

n = 9,022

MDC group before matching

n = 1,065

Fee for guidance within 1 year of the index date

(MDC interval ≤365 days^a^)

n = 1,065

Fee for guidance

after 1 year from the index date

(MDC interval >365 days)

n = 439

No fee for guidance within observation period

n = 8,583

Eligible patients

n = 10,087

Patients with diabetes prescriptions in the RWD database

n = 484,635

T2DKD diagnosis

n = 66,316

Excluded n = 418,319

No T2DKD diagnosis

(C)

Excluded n = 56,229

Under 19 years old (n = 153)

Index eGFR <15 or >90 mL/min/1.73 m^2^ or no eGFR data

(n = 42,395)

Diagnosis history of T1DM (n = 3,170)

History of KRT before or within 1 year of the index date (n = 309)

Follow-up observation period less than 1 year or study period before April 2012 (n = 8,127)

No T2DM diagnosis before the index date (n = 1,573)

History of MDC before the index date (n = 72)

KFRT occurred within the landmark time (n=430)

^a^ Duration between diagnosis of diabetic nephropathy and the first guidance to prevent dialysis for patients with diabetes.

RWD: Real World Data, T2DKD: type 2 diabetic kidney disease, eGFR: estimated glomerular filtration rate, T1DM: type 1 diabetes mellitus, KRT: kidney replacement therapy, T2DM: type 2 diabetes mellitus, MDC: multidisciplinary care, KFRT: kidney failure with replacement therapy

**Supplemental Figure 5**

Kaplan–Meier curves for the main analysis of secondary outcomes: (A) death, (B) permanent dialysis, (C) hospitalization, (D) temporary catheterization, and (E) KFRT


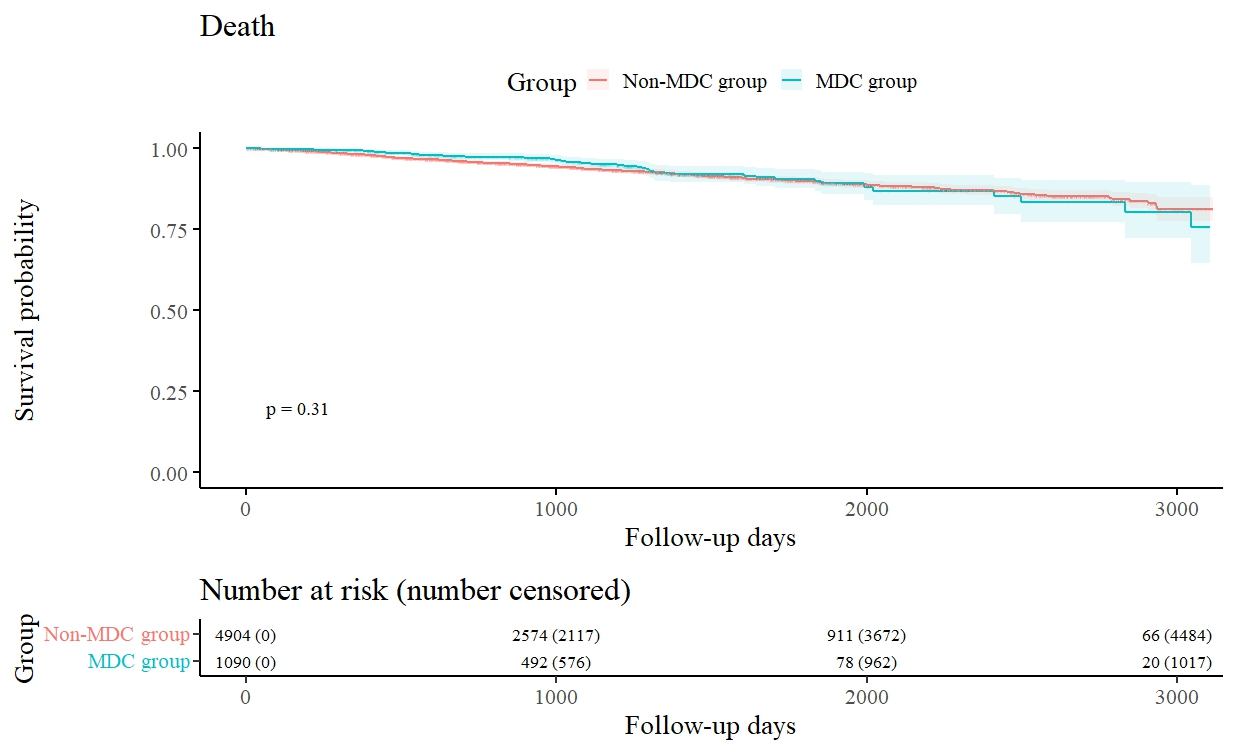


**(A)**


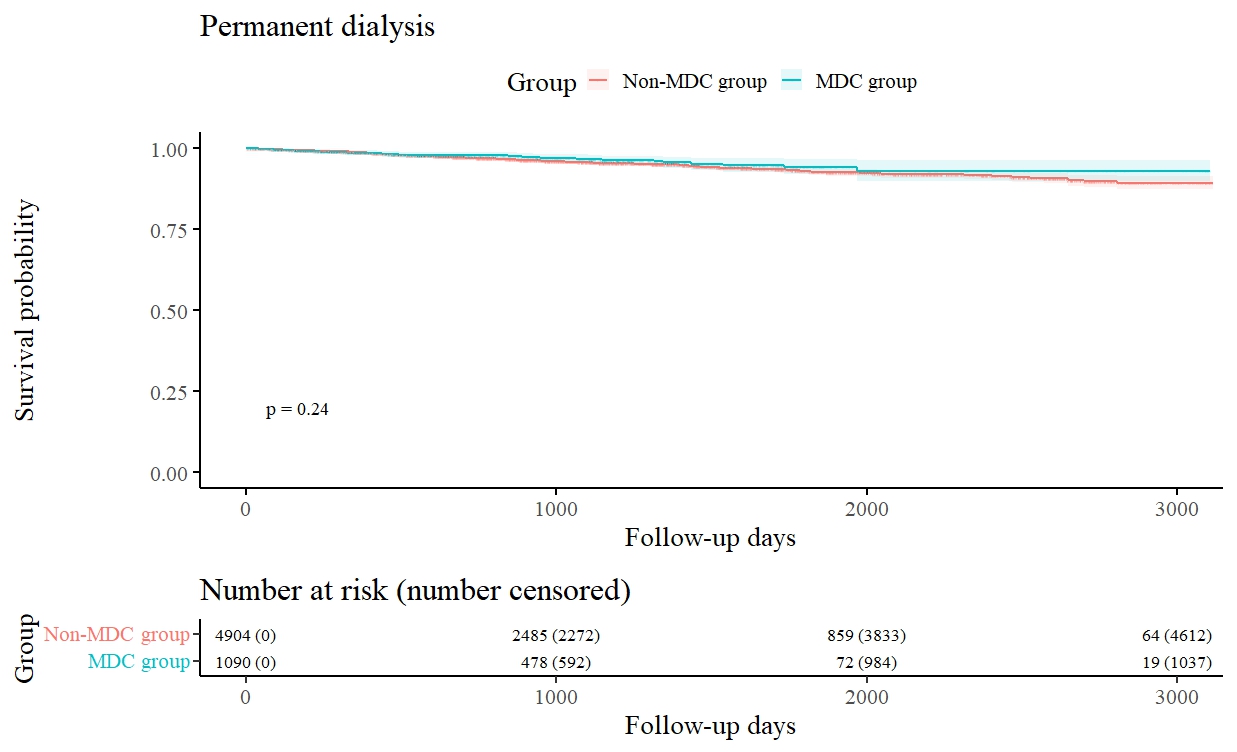


**(B)**

**(C)**


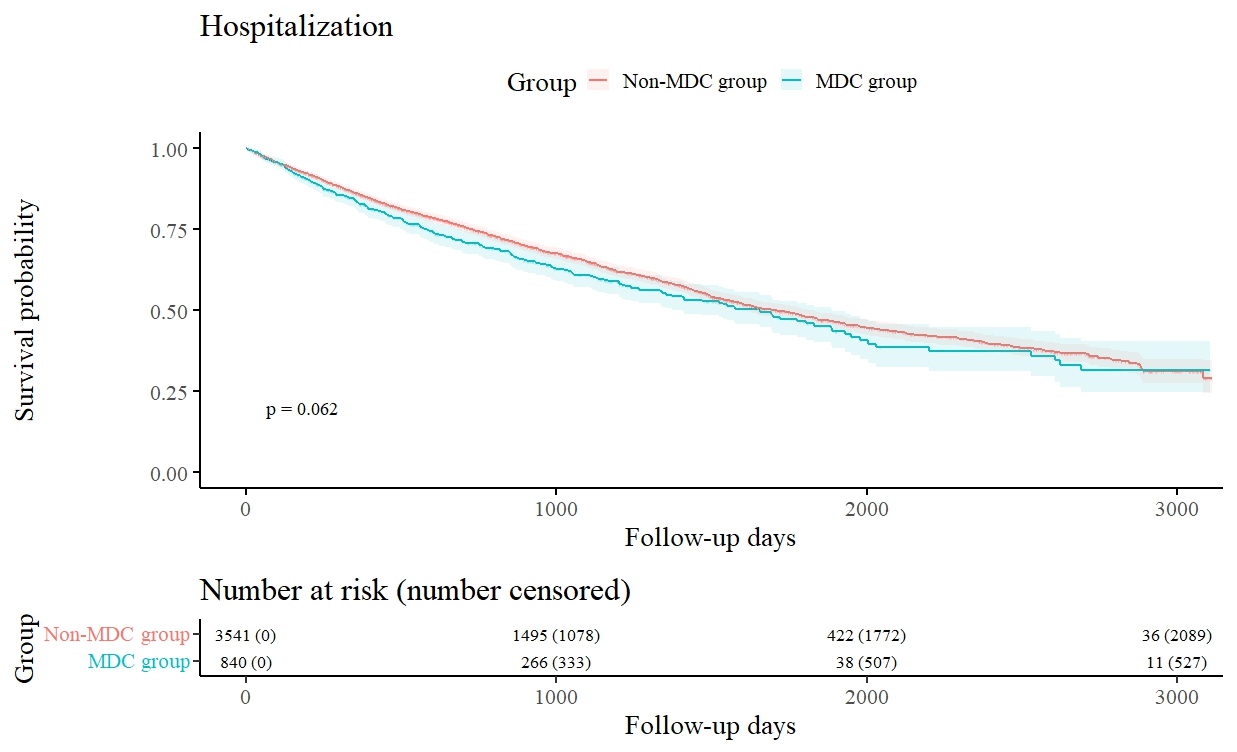

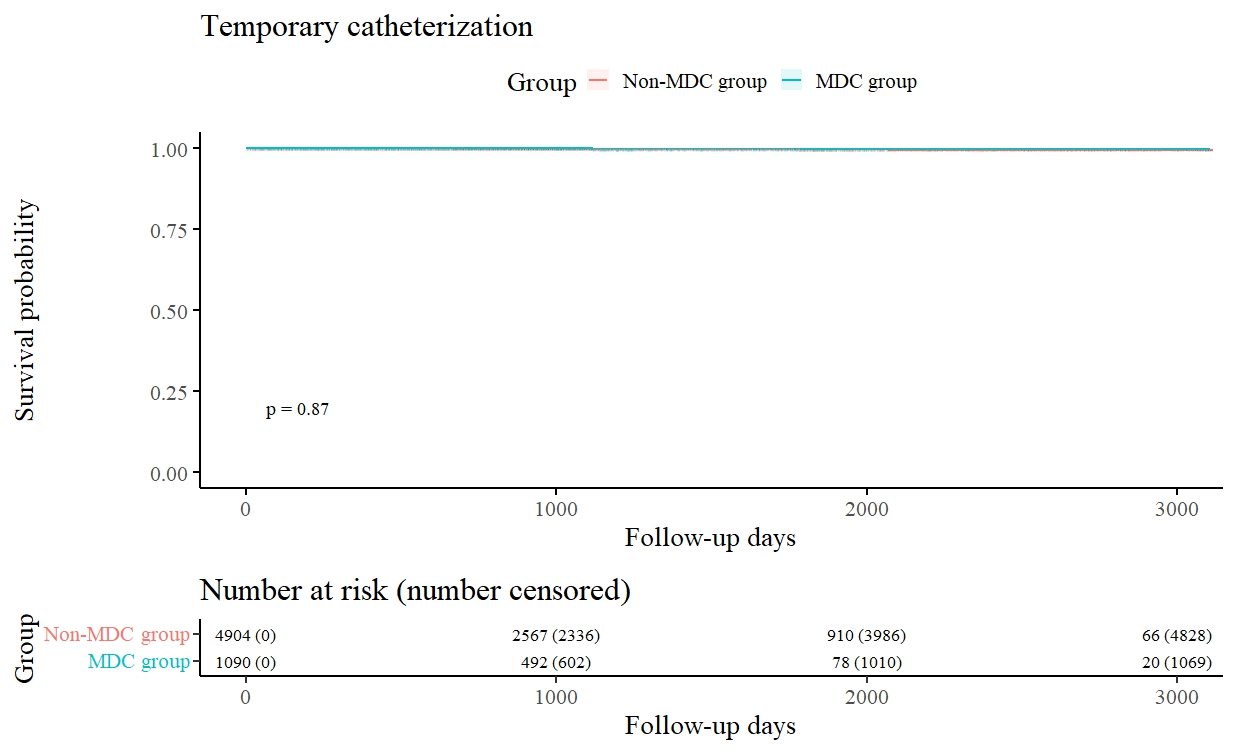


**(D)**


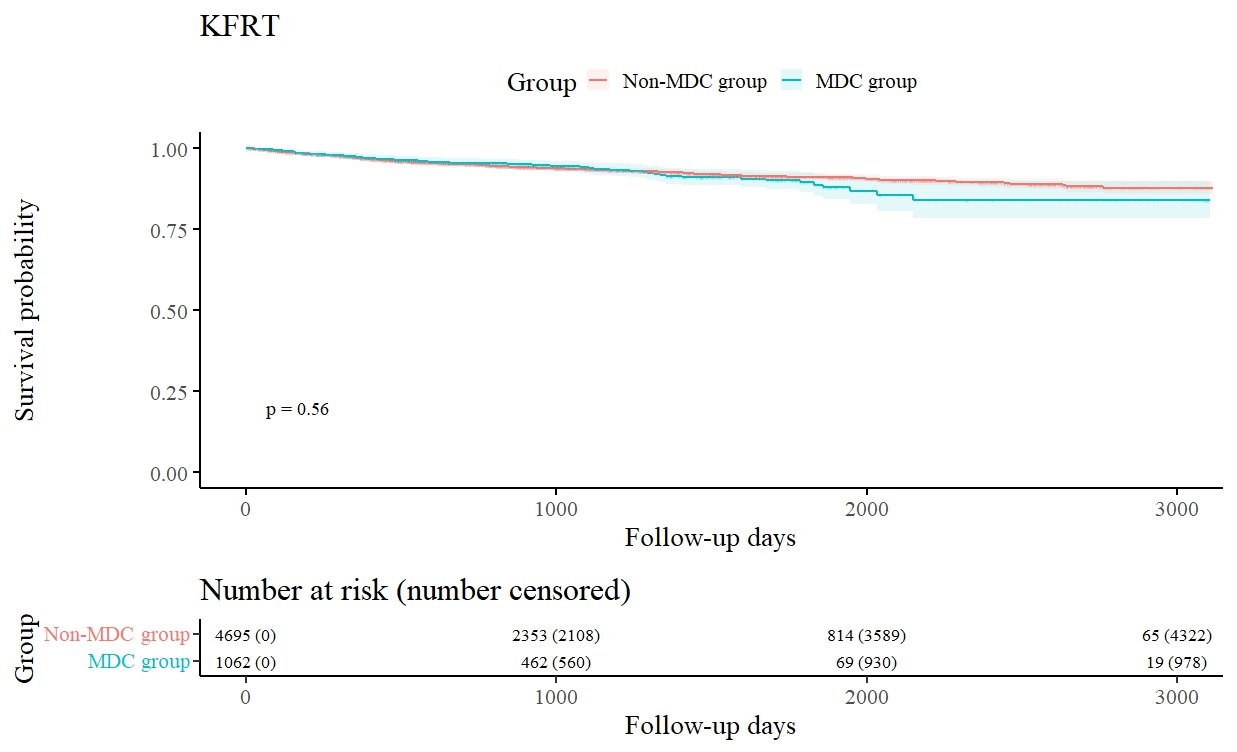


**(E)**

The survival probabilities of the secondary outcomes were calculated using the Kaplan–Meier method. The log-rank test was used to calculate P values.

MDC: multidisciplinary care, KFRT: kidney failure with replacement therapy
